# RFEA-Derived Ion-Energy Descriptors for Preliminary Process-Window Evaluation in Dual-Beam Soft Plasma Etching of Organic Semiconductor Films

**DOI:** 10.3390/mi17101139

**Published:** 2026-09-30

**Authors:** Rui Gao, Mengna Dai, Xin Zou, Yuheng Wang

**Affiliations:** Department of Medical Engineering, Second Affiliated Hospital, Army Medical University, Chongqing 400030, China

**Keywords:** soft plasma etching, ion-energy-distribution function, RFEA, organic semiconductor, dual-beam modulation, X-ray photoelectron spectroscopy, atomic force microscopy, candidate-condition ranking

## Abstract

Low-damage plasma processing of organic semiconductor films requires a consistent way to relate plasma diagnostics to material responses. This study presents a retarding-field energy analyser (RFEA)-guided workflow for preliminary evaluation of seven nominal single- and dual-beam settings. A selectivity-ratio (SR) screening index was calculated as the ratio of integrated ion-energy-distribution intensity in fixed 4–6 eV and 6–8 eV windows. The seven recorded scores range from 1.32 to 7.58, with the 50 + 100 condition label giving the highest window ratio. The index describes the relative spectral weight of the two intervals under an assumed activation-versus-damage interpretation. A published effective-energy model provides separate physical context. Time-resolved optical measurements and baseline electrical characterisation were complemented by X-ray photoelectron spectroscopy (XPS) and atomic force microscopy (AFM). At 100 Pa for 100 s, dual-beam treatment showed higher mean surface O/C while retaining mean roughness close to the unetched films. Additional stylus profilometry comprised 42 scans from 14 specimens. At 50 Pa for 180 s, treated-group mean apparent step heights ranged from 160.7 to 205.9 nm, without a uniform increase under source superposition. These within-protocol measurements provide complementary criteria for assessing candidate conditions. The workflow combines an explicit spectral ranking with measured film responses for preliminary process-window evaluation; SR is interpreted as a screening index rather than a measured molecular etch-rate ratio.

## 1. Introduction

Organic semiconductor thin films are central to flexible and plastic electronics, or-ganic photovoltaics, solution-processed transistors, and biointegrated electronic systems [1,2,3,4,5,6,7,8,9,10]. In these films, molecular packing, compositional gradients, dopant distributions, and electronic functionality can vary along the film-normal direction; consequently, the near-surface layer need not represent the transport-relevant bulk or buried interface [11,12,13,14,15,16,17,18,19,20]. This vertical heterogeneity creates an opportunity for controlled surface removal, but also makes the processing of organic semiconductors particularly sensitive to exces-sive ion bombardment, oxidation, and disruption of conjugated molecular order.

Soft plasma processing has therefore attracted interest as a route to selectively modify or incrementally remove organic semiconductor surface layers while retaining useful subsurface structure and device function [16,17,18,19,20,21,22,23,24,25,26]. Previous studies have used low-pressure soft plasma etching and spectroscopic depth-profiling strategies to examine film-depth-dependent composition and electronic behaviour in organic thin films [16,17,18,19,20]. However, useful process development requires more than a nominal source setting: it requires a physically meaningful description of the plasma state, together with direct structural and chemical validation of the corresponding film response.

More broadly, low-temperature plasma processing is governed by coupled ion, electron, neutral-radical, photon, and surface-reaction pathways [27,28,29,30]. Ion-energy distributions at the substrate are particularly important because they connect sheath dynamics with the balance between activation, desorption, bond scission, and damage [31,32,33,34,35,36]. Tailored substrate-bias waveforms have been shown to reshape ion-energy distributions and, in suitable operating regimes, provide additional control over the energy delivered to the surface [34,35,36]. For chemically vulnerable organic films, this provides the motivation to monitor plasma-state descriptors rather than relying on a single nominal control amplitude.

Multi-frequency and tailored-voltage studies show how plasma control can modify ion flux and energy [37,38,39,40,41,42,43,44,45,46,47,48,49,50,51,52,53]. They motivate diagnostic measurements when comparing source settings. Earlier soft-plasma work reported a CNN–LSTM spectral-prediction network and a separate physical response relation [21]. The present study uses that relation as model context. No machine learning training or inference was performed here.

**Figure 1:**
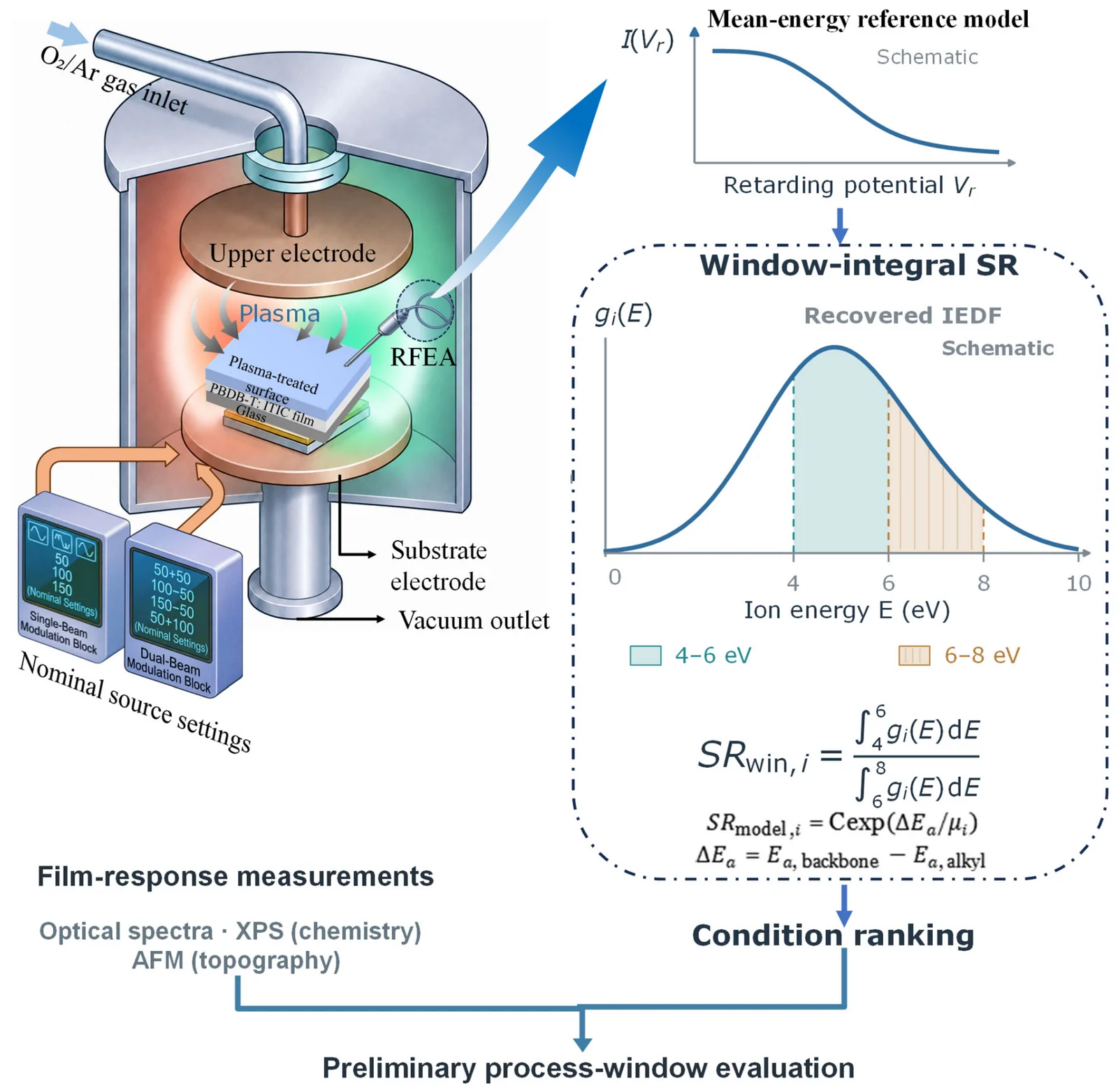
Schematic of the RFEA-guided workflow for preliminary process-window evaluation in soft plasma etching of PBDB-T films. Collector current–voltage sweeps acquired using a retarding-field energy analyser (RFEA) are processed to recover the ion-energy distribution (*g*_i_(*E*)) [54]. The shaded 4–6 eV and 6–8 eV intervals define the numerator and denominator of the window-integral screening index (*SR*_win,*i*_), respectively. All conditions use identical integration bounds and are ranked by descending (*SR*_win,*i*_). The exponential expression provides a separate mean-energy reference model [21] and does not determine this ranking. Here, (*μ_i_*) denotes the mean ion energy, (C) is a common response-prefactor ratio, and (Δ*E_a_*) is the assigned activation-energy difference. Optical spectra, X-ray photoelectron spectroscopy (XPS) and atomic force microscopy (AFM) characterise film evolution, surface composition and topography, respectively, providing complementary criteria for process-window evaluation. The two energy windows are operational spectral weights under the stated activation-versus-damage assumptions. The chamber geometry, control blocks and plotted curves are schematic.

The objective of this work is to define RFEA-guided SR screening for preliminary process-window evaluation. We specify the two-window calculation and its response assumptions, then examine optical, XPS, AFM and profilometry results that bear on process selection. Single-beam modulation denotes one emitter operating, and dual-beam-superimposed modulation denotes two emitters operating simultaneously. Figure 1 presents the diagnostic concept and the mean-energy reference model. Section 2.3 distinguishes that reference model from the window-integral index used to rank the seven conditions.

## 2. Experimental Section

### 2.1. Materials and Thin-Film Preparation

Organic semiconductor blend films (PBDB-T: ITIC, donor: acceptor = 1:1 by mass) (PBDB-T, Organtec Ltd., Beijing, China; ITIC, Solarmer Materials Inc., Beijing, China) were spin-coated onto pre-cleaned glass substrates at 2000 rpm to a nominal thickness of 100 nm, following the protocol described in Ref. [21]. The initial film thickness was checked by profilometry. The films provided the material-response measurements accompanying the RFEA-guided screening framework, including time-resolved optical monitoring during film formation and plasma treatment, baseline electrical characterisation before etching, and XPS and AFM analysis of selected unetched, single-beam-treated, and dual-beam-superimposed samples. Optical, baseline electrical and surface measurements are described below; supporting specimen records are given in Supplementary Sections S1 and S7–S9.

### 2.2. Plasma Modulation Settings

Plasma treatment used an inductively coupled plasma (ICP) system. The reported feed gas was O_2_/Ar (80:20 *v*/*v*). Figure 1 shows the conceptual chamber and its RFEA diagnostic. The spectroscopic apparatus used for optical acquisition is described in Section 2.4.

Single-beam modulation denotes one emitter operating, and dual-beam-superimposed modulation denotes two emitters operating simultaneously. Ordered source powers are retained, so 50 W + 50 W means 50 W at each source. Superposition refers to concurrent operation. Emitter positions, drive frequency, duty cycle and relative phase were not established in the available records, so phase-controlled excitation is not assumed. Nominal source power is reported separately from ion energy.

Figure S4 and Table S11 preserve the controller display. Its cycle-time and delay fields do not establish RF settings or alter the reported treatment durations.

Optical acquisition used 50 Pa, 50 W and 100 s, with 1 s sampling. XPS and AFM compare unetched films and films treated at 100 Pa for 100 s at 50 W or 50 W + 50 W (Table S12). These settings change both source number and nominal total power, so their separate effects cannot be isolated.

The separate profilometry series used 50 Pa and 180 s. Table S6 gives the seven ordered source settings and specimen assignments. These specimen numbers are distinct from the AFM film IDs in Table S1.

### 2.3. RFEA Descriptors and Window-Integral SR

RFEA characterises ion energy through collector current recorded across a retarding-potential sweep [54]. Recovering an energy profile requires the voltage reference, energy conversion, background and analyser response. Probe grids and electric fields can affect low-energy transmission [55]. RFEA-derived profiles stored in MATLAB 2022 .mat format were processed using a window-integral ratio, and the resulting SR values were recorded in Density_summary.csv. The seven scores were read from that summary without recalculation during plotting. Supplementary Sections S2 and S12 describe the file records and calculation definition.

SR denotes the two-window screening index, written as *SR_win_*_,*i*_ below. The subscript model identifies a score under the response formulation specified with each reference equation. Let *g_i_*(*E*) denote a nonnegative, flux-weighted energy profile at the sample position for condition i. Its mean energy is calculated over the full measured range using the recorded energy spacing.

(1) $$\mu_{i}=\frac{\int \;Eg_{i}(E)\;\mathrm{dE}}{\int g_{i}(E)\;\mathrm{dE}}$$

The distribution SD is the square root of the second central moment. FWHM is obtained from interpolated half-height crossings when one connected peak region and both crossings are resolved. These descriptors describe profile breadth. The operational SR uses the profile intensities in two fixed energy windows, applied consistently across the seven conditions.

(2) $${\mathrm{SR}}_{\mathrm{win},i}=\frac{\int_{4}^{6}g_{i}(E)\;\mathrm{dE}}{\int_{6}^{8}g_{i}(E)\;\mathrm{dE}}$$

The integration limits are in eV. A higher *SR_win_*_,*i*_ indicates greater intensity in 4–6 eV relative to 6–8 eV. Both window integrals must be resolved, and the denominator must be positive. A common multiplicative intensity normalisation cancels in the ratio. Ranking is based on descending *SR_win_*_,*i*_, not on mean energy or FWHM alone. The two windows define the operational weighting used here; assigning the lower interval to favourable activation and the upper interval to competing damage is a screening assumption, not a measurement of chemically exclusive reaction channels. Intensity outside 4–8 eV does not enter this ratio.

The published effective-energy relation [21] is proportional to an exponential involving the difference between the assigned backbone and alkyl activation energies. For comparison with this prior physical interpretation, we retain the following mean-energy reference model, with Δ*E_a_* = Ea, backbone −* E_a_*, alkyl.

The reference approximation evaluates two effective response factors at the mean ion energy. Each factor is represented by a response prefactor multiplied by an exponential of minus its assigned activation energy divided by that mean energy. Taking their ratio gives Equation (3). This relation describes a phenomenological response scenario under a fixed effective ion population and comparable surface response. It provides theoretical context for selective processing; it is not the algorithm used to generate the seven window-integral scores. The numerical 4–6 eV and 6–8 eV windows are the definitions adopted in this study and are not attributed to Ref. [21].

(3) $${\mathrm{SR}}_{\mathrm{model},i}=C\;\exp(\frac{{\Delta E}_{a}}{\mu_{i}})$$

Equation (3) represents an assumed differential response through a phenomenological screening relation. The dimensionless factor C is the ratio of the two response prefactors under a common response convention. It determines the absolute scale and requires an independent basis. Setting C to one does not establish absolute selectivity. When both the activation-energy difference and a common positive C are held fixed across conditions, the model can instead be expressed relative to a declared reference condition *r*.

(4) $$R_{i}=\frac{{\mathrm{SR}}_{\mathrm{model},i}}{{\mathrm{SR}}_{\mathrm{model},r}}=\exp[{\Delta E}_{a}(\frac{1}{\mu_{i}}-\frac{1}{\mu_{r}})]$$

*R_i_* is the ratio to the reference condition and is labelled relative SRmodel in the calculation package. For positive Δ*E_a_* and a common C, this reference model ranks conditions through mean energy. SD and FWHM describe profile shape and inform separately stated sensitivity scenarios. The positive-width branch used to reproduce the archived analytical curves is reported in Supplementary Section S5 as a distinct model scenario.

Within Equations (3) and (4), descending reference-model SR gives the same order as ascending mean energy for shared positive parameters. This mathematical property does not constrain *SR_win_*_,*i*_ in Equation (2). Profiles with the same mean may distribute different intensities between the two windows and therefore give different window scores. Supplementary Section S10 shows how the window ratio is represented using interval-weighting functions in a general profile-integral formulation. Quantitative reaction-yield functions would require additional physical support.

### 2.4. Time-Resolved Optical and Baseline Electrical Characterisation

**Figure 2:**
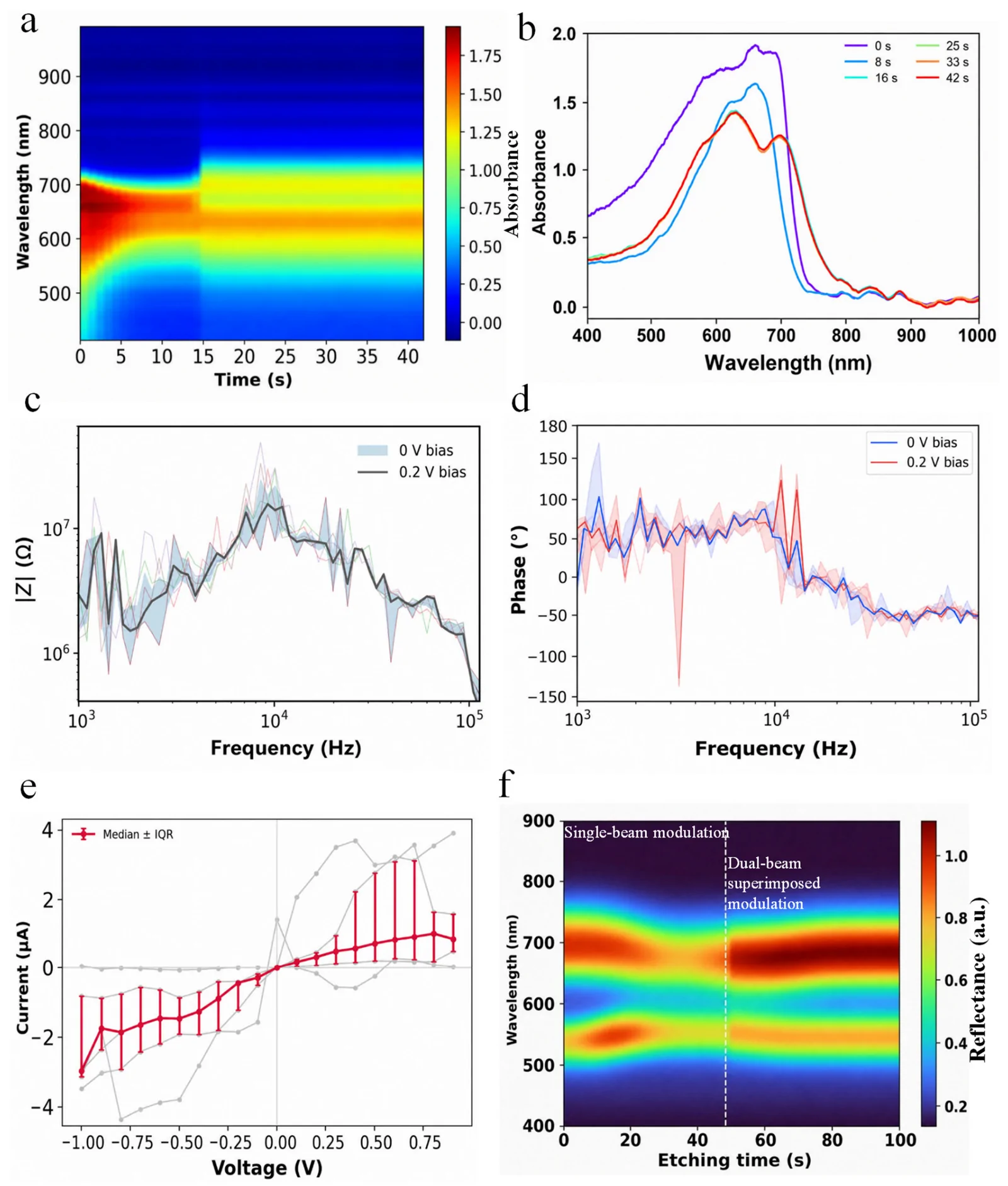
Optical measurements during etching and baseline electrical characterisation of PBDB-T:ITIC films. Optical acquisition used 50 Pa, a nominal setting of 50 W, a 100 s treatment protocol and 1 s sampling. (**a**) Early-time absorbance map. (**b**) Selected spectra at 0, 8, 16, 25, 33 and 42 s. (**c**) Impedance magnitude |Z| and (**d**) phase angle of the unetched film at nominal DC biases of 0 and 0.2 V. (**e**) Baseline-corrected I–V scans of the same unetched film. Grey traces are technical repeats; the red curve and error bars indicate the pointwise median and interquartile range. (**f**) Reflection map labelled with single-beam (0–50 s) and dual-beam-superimposed (50–100 s) intervals. The dashed line marks the interval change. Time is not calibrated to depth, and the second interval includes cumulative exposure from the first.

Absorbance and reflection spectra were acquired during plasma etching using a xenon lamp in a darkened environment and the film-depth spectroscopy apparatus developed at Xi’an Jiaotong University. The protocol was 50 Pa, 50 W and 100 s, with spectra sampled every 1 s. Absorbance was expressed as *A *= −*log*_10_(*I*/*I*_0_). Figure 2a shows the early etching interval, and Figure 2b presents spectra at 0, 8, 16, 25, 33 and 42 s. The 1 s interval describes the sampling cadence; the available acquisition records do not specify the detector integration time or the detailed reference-correction procedure.

The frequency response of the unetched films was measured using a Tonghui TH2840B precision LCR metre (Tonghui Electronic, Changzhou, China) at nominal DC biases of 0 and 0.2 V. Impedance magnitude |Z| and phase were recorded over 10^3^–10^5^ Hz. Individual traces, their central trend and dispersion describe the baseline electrical response and scan repeatability. Microscope-assisted contacts used MyProber PM-004A probe holders (MyProber, Guangzhou, China) (Figure S5).

Sequential current–voltage scans were obtained from the same unetched film over −1.0 to 0.9 V. Baseline-drift correction was applied before calculating the pointwise median and interquartile range. The dispersion describes technical repeatability of one film, not variation among independently prepared films or devices.

The reflection map in Figure 2f covers the 100 s sequence at the same pressure, nominal power setting and sampling interval. Its interval labels identify single-beam modulation during 0–50 s and dual-beam-superimposed modulation during 50–100 s. The second interval begins with an already exposed film, so its response includes the effect of cumulative treatment. Elapsed time is retained as the map coordinate because no independent time-to-depth calibration is available.

### 2.5. Statistical Analysis

The analysis distinguishes independent films or specimens from repeated measurements of one sample. Means and sample SDs summarise film- or specimen-level values. For RFEA, profile SD and FWHM describe distribution breadth. Uncertainty in mean energy instead requires acquisition or calibration information. Model-parameter scenarios quantify deterministic sensitivity and carry no statistical confidence level.

Optical and electrical data were analysed descriptively. XPS results are means ± SD across two films per condition. For AFM, three regions were averaged within each film before calculating means and sample SDs across two films. For profilometry, three scans were averaged within each treated specimen, followed by group statistics across specimen means. The 50 W + 100 W condition has one specimen, so a between-specimen SD is not estimable. The thickness estimate from specimen 14 uses one confirmed scratch location. It has no between-location SD and is excluded from the etching-condition comparison. No inferential test was applied.

### 2.6. X-Ray Photoelectron Spectroscopy

X-ray photoelectron spectroscopy was performed by an external testing service using a Thermo Scientific K-Alpha instrument (Thermo Fisher, Shanghai, China). Two independently prepared films were analysed for each condition: unetched, single-beam-modulated (100 Pa, 50 W, 100 s), and dual-beam-superimposed (100 Pa, 50 W + 50 W, 100 s) films. Survey spectra and high-resolution C 1s, O 1s, N 1s, S 2p, and Si 2p spectra were collected without Ar^+^ sputter cleaning. Surface atomic concentrations were calculated from the survey spectra using the instrument sensitivity factors and are reported as mean ± standard deviation for the two independently prepared films. The high-resolution spectra were background-subtracted and independently normalised for qualitative comparison of their line shapes. Because constrained component fitting and a unified quantitative peak-shift analysis were not performed, the high-resolution spectra were not used to calculate chemical-state fractions or to make quantitative binding-energy-shift claims.

### 2.7. Atomic Force Microscopy

**Figure 3:**
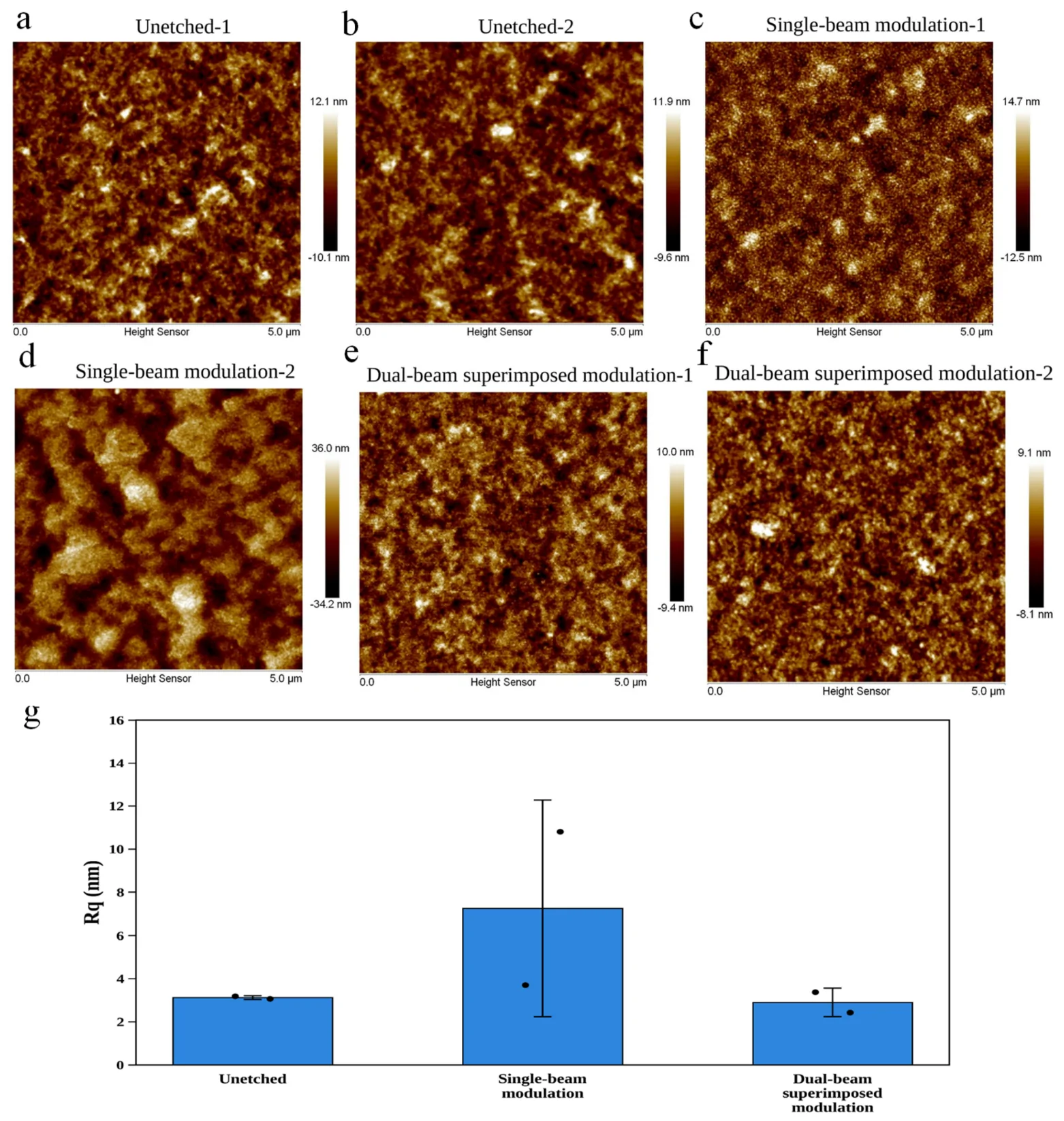
AFM surface morphology and roughness analysis of PBDB-T: ITIC films under different plasma-modulation conditions. (**a**,**b**) AFM height images of two independently prepared unetched films, U−1and U−12. (**c**,**d**) AFM height images of two independently prepared films after single-beam modulation, E50-2-1 and E50-2-2. (**e**,**f**) AFM height images of two independently prepared films after dual-beam-superimposed modulation, E50-1 and E50-2. All images were acquired over an area of 5 μm × 5 μm. Each image is displayed using its own height scale, as indicated by the corresponding colour bar; therefore, quantitative cross-condition comparisons should be based primarily on the roughness values rather than colour contrast alone. (**g**) Root-mean-square roughness (Rq) of the unetched, single-beam-modulated, and dual-beam-superimposed films. Three regions were analysed for each film and averaged to obtain one independent-film value. Bars represent the mean ± standard deviation of two independently prepared films per condition (n = 2), and black dots denote the individual-film means. The corresponding Rq values were 3.12 ± 0.09 nm, 7.25 ± 5.02 nm, and 2.90 ± 0.66 nm, respectively. The single-beam condition exhibited a larger but markedly sample-dependent roughness response.

Surface topography was characterised by an external testing service using a Bruker Dimension Icon atomic force microscope (Bruker, Billerica, MA, USA). Two independently prepared films were analysed for each condition: unetched, single-beam-modulated (100 Pa, 50 W, 100 s), and dual-beam-superimposed (100 Pa, 50 W + 50 W, 100 s). Three 5 μm × 5 μm regions were acquired from each film. Root-mean-square roughness (Rq) and arithmetic mean roughness (Ra) were extracted from the processed height images supplied by the instrument software. The three region-level values were averaged to obtain one value for each independent film, and group results were reported as mean ± standard deviation across the two films. One representative height image from each film is displayed in Figure 3. The original sample identifiers and treatment assignments are documented in Supplementary Table S1.

### 2.8. Stylus Profilometry Across Etched and Unetched-Film Regions

The profilometry series comprised 14 specimens, each with three scans. Specimens 1–13 were treated at 50 Pa for 180 s. Specimen 14 was the unetched-film thickness reference. Table S6 maps the specimen IDs to their settings and roles. The order of the superimposed power components is retained, so 50 W + 100 W and 100 W + 50 W are distinct settings. These IDs apply only to profilometry.

For specimens 1–13, three scans crossed the boundary between etched and unetched-film regions. The measurement request specifies upper, middle and lower positions, although the exported CSV files do not identify the exact position of each scan. In the unetched reference, specimen 14, the trough at approximately 796–816 µm in scan 14-3 was an intentional scratch reaching bare glass; its shoulders were unetched film. Supplementary Sections S7.4 and S8 document the reference geometry and supporting records.

Each scan contains 5401 points over a nominal 900 µm length, acquired in 18 s at 0.166605 µm intervals, with a 2 µm stylus radius and a 3 mg force setting. Heights in Å were divided by 10 to obtain nm. Original levelling, offsets and all points were retained. Only the scratch-thickness calculation uses a local baseline.

For specimens 1–13, the apparent step height Δh was calculated as the median height at 700–800 µm minus that at 50–150 µm. All values were positive in the exported scan direction. For scan 14-3, local thickness was the median separation of the scratch floor at 800–812 µm from a line interpolated through film-shoulder medians at 775–790 and 825–840 µm. The common-window differences for specimen 14 were retained as diagnostics and were not subtracted from the treated values. Supplementary Section S7 contains all 42 profiles and the analysis definitions.

## 3. Results and Discussion

### 3.1. Window-Integral SR Ranking and Model Interpretation

The reported condition ranking uses the window-integral SR defined in Equation (2). The analytical curves in Supplementary Section S5 separately illustrate the published effective-energy relation and a declared width-sensitivity scenario. Equations (3) and (4), the positive-width branch in Equation (S1) and the general response-integral form in Equation (S4), are retained to distinguish the assumptions behind different interpretations of SR. The fixed-window specialisation of Equation (S4) gives the operational index used here.

For positive Δ*E_a_* and fixed C, the reference-model score in Equation (3) decreases as mean energy increases. The two-window ratio in Equation (2) instead depends on the relative areas within the specified intervals. Its order can differ from the reference model without an algebraic inconsistency. Neither ratio alone determines the amount of material removed, so process selection also considers surface recession and the remaining film response.

The seven recorded window scores span 1.32–7.58. Their descending order is 50 + 100, 50 + 50, 100−50, 150−50, 50, 100 and 150, using the original row labels in Table S2. The 50 + 100 label has the highest score, 7.5809155, and therefore the largest recorded ratio of 4–6 eV to 6–8 eV intensity. This identifies the leading condition under the chosen spectral criterion, not an experimentally established optimum in molecular selectivity. Section S11 explains why these scores should not be reconstructed from the archived mean and signed-width columns using the exponential reference relation.

The following sections present the measured film responses within each experimental protocol. Profilometry assesses apparent surface recession, optical spectra follow film evolution, and XPS and AFM examine the remaining surface. These observations supply complementary process criteria. Their different protocols do not provide a condition-by-condition validation of all seven SR scores.

### 3.2. Etched-to-Unetched Step Heights in the 50 Pa Specimen Set

**Figure 4:**
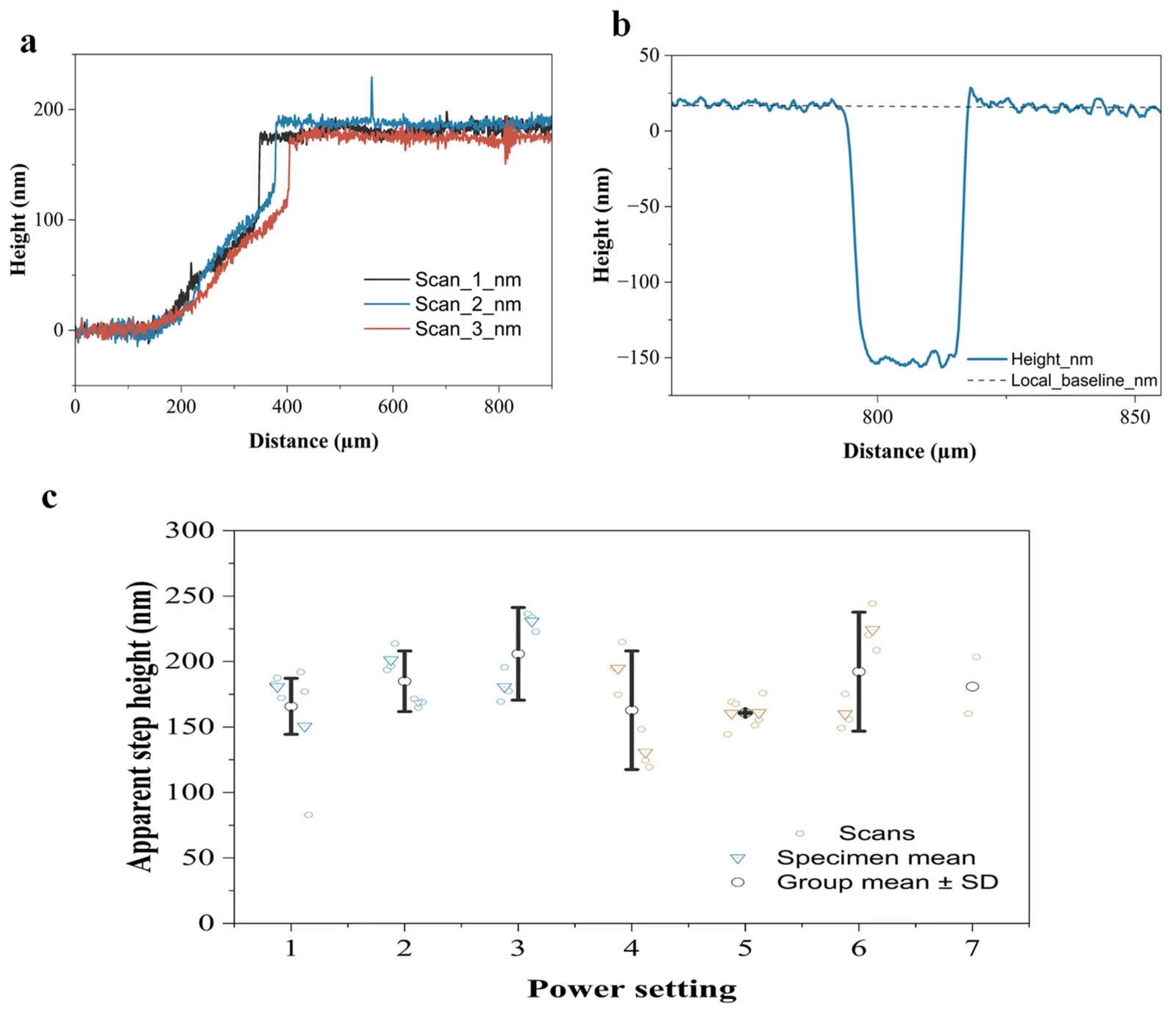
Profilometry of treated films and the unetched reference. (**a**) Three etched/unetched boundary scans for specimen 1 (50 W, 180 s). The step-analysis windows are defined in Section S7. (**b**) Confirmed glass-exposing scratch in scan 14-3. The dashed line joins the film-shoulder medians. Its median separation from the scratch floor gives a local initial thickness of 168.6 nm at one location. The shoulder and floor windows are specified in Section S7.4. Original offsets and full profiles are retained in the SI. (**c**) Apparent step heights for specimens 1–13. Small open circles denote scans, open triangles denote specimen means, and larger open circles denote group means with black sample-SD error bars where n = 2. Power-setting indices 1–7 follow Table 1; blue and orange identify single-source and superimposed settings. Specimen 14 is excluded.

Figure 4 shows representative etched/unetched steps, the local unetched-film thickness reference and treated-condition summaries. Single-source settings of 50, 100 and 150 W gave mean Δh values of 165.7 ± 21.3, 184.9 ± 23.2 and 205.9 ± 35.4 nm. The corresponding 50 W + 50 W, 100 W + 50 W and 150 W + 50 W groups gave 162.8 ± 45.3, 160.7 ± 0.4 and 192.3 ± 45.5 nm. Each mean and sample SD uses two specimen means. The single 50 W + 100 W specimen gave 181.0 nm. Scan 14-3 gave a local unetched-film thickness of 168.6 nm. Window-placement sensitivity is reported in Section S7.4. All scans and summaries are provided in Figures S2 and S3 and Tables S7 and S8.

**Table 1:** Etched-to-unetched step heights for the seven treated conditions at 50 Pa. Values are in nm. Each specimen mean uses three scans. Group means and sample SDs use specimen means; n: specimen counts. Between-specimen SD is not estimable when n = 1. The unetched-thickness reference, specimen 14, is assessed separately in the SI.

Power Setting	Specimen IDs	n	Mean Δh	SD Across Specimens
50 W	1, 2	2	165.7	21.3
100 W	3, 4	2	184.9	23.2
150 W	5, 6	2	205.9	35.4
50 W + 50 W	7, 8	2	162.8	45.3
100 W + 50 W	9, 10	2	160.7	0.4
150 W + 50 W	11, 12	2	192.3	45.5
50 W + 100 W	13	1	181.0	Not estimable

Superimposed settings did not give uniformly larger height differences. Within-specimen variation was appreciable: sample 2 gave 192.0, 177.0 and 82.9 nm across its three scans. Conversely, the two similar specimen means at 100 W + 50 W produced a small group SD despite scan-level variation. Changing the common analysis windows altered specimen means by ranges of 0.5–10.1 nm (Tables S9 and S10). This assesses window placement, not total measurement uncertainty.

The etched/unetched steps provide geometric evidence of apparent surface recession. The local unetched thickness of 168.6 nm differs from the nominal 100 nm preparation value, and several treated-specimen mean steps, reaching 230.9 nm, exceed this local reference. Assigning the full step to organic-film removal therefore requires initial and residual thickness measurements on the corresponding specimens. The reported quantity is apparent step height, without a thickness-normalised removal fraction or component-specific selectivity.

### 3.3. Optical Evolution During Plasma Etching

The measurement sets address different aspects of film response. Profilometry uses 50 Pa and 180 s, optical monitoring uses 50 Pa and 100 s, and XPS/AFM uses 100 Pa and 100 s. Comparisons are therefore made within each protocol. Profilometry specimen numbers and AFM film identifiers refer to separate sample sets. Section 3.6 discusses how these observations inform process evaluation.

The absorbance map shows a transition near 14–16 s, followed by a more stable pattern across the broad 600–700 nm region (Figure 2a). The selected spectra show the same temporal change (Figure 2b). The optical response can reflect changes in thickness, composition, molecular order and scattering. It therefore records film evolution during exposure without resolving the removal of individual molecular components.

The unetched film showed a broad impedance maximum near 10^4^ Hz, followed by a decrease at higher frequencies (Figure 2c). Its phase was predominantly positive below approximately 10^4^ Hz and negative at higher frequencies (Figure 2d). The 0 and 0.2 V bias conditions had similar overall trends, with appreciable dispersion among scans. These traces establish the baseline frequency response for the measured film.

For the repeated I–V scans of the same unetched film, median current changed from negative to positive as the voltage crossed 0 V (Figure 2e). The interquartile range widened at larger voltage magnitudes. The grey traces show variability among sequential scans, potentially associated with contact evolution, charge accumulation or instrumental drift. The summary describes the technical repeatability of this film.

The reflection map changes across the two treatment intervals, including features near 530–570 and 650–720 nm (Figure 2f). The change accompanies the switch at 50 s. Since the second interval starts from an already exposed film, the sequence combines source configuration with cumulative treatment. It provides a time-resolved record of optical evolution.

### 3.4. Surface-Composition Changes Measured by XPS

**Figure 5:**
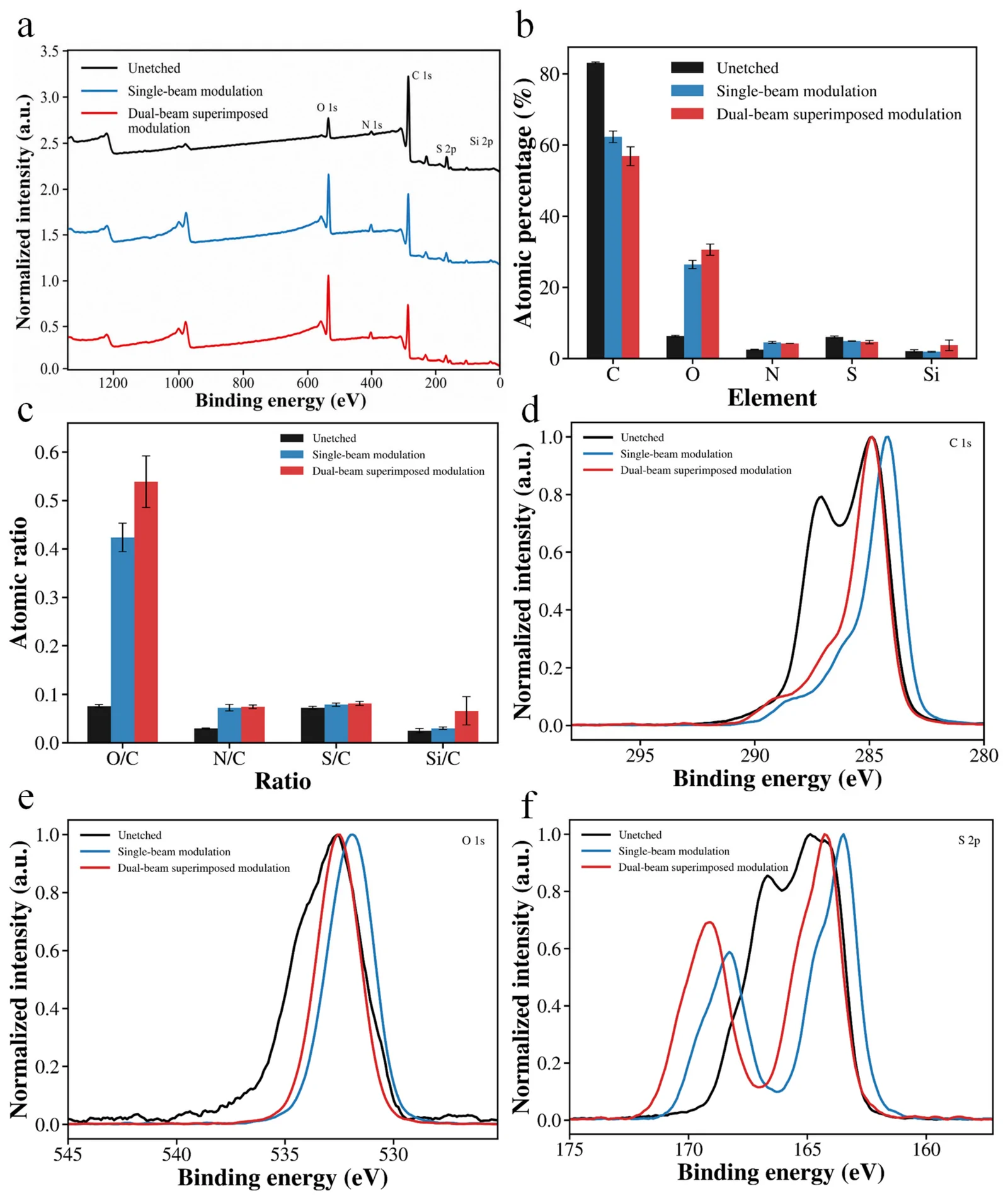
XPS surface characterisation of unetched, single-beam-modulated and dual-beam-superimposed PBDB-T:ITIC films. The treated groups used 100 Pa for 100 s, with nominal settings of 50 W and 50 W + 50 W, respectively. (**a**) Survey spectra. (**b**) Surface atomic percentages of C, O, N, S and Si. (**c**) O/C, N/C, S/C and Si/C atomic ratios. Bars show means and SDs for two independently prepared films per condition (n = 2). (**d**–**f**) Background-subtracted, independently normalised high-resolution C 1s, O 1s and S 2p spectra, respectively. The high-resolution spectra are compared qualitatively; fitted chemical-state fractions are not reported.

XPS shows a lower carbon fraction and a higher oxygen fraction after plasma treatment (Figure 5a–c; Table 2). Carbon decreased from 83.11 ± 0.23 at.% in unetched films to 62.31 ± 1.63 at.% after single-beam modulation and 56.84 ± 2.64 at.% after dual-beam-superimposed modulation. Oxygen increased from 6.31 ± 0.23 at.% to 26.39 ± 1.15 and 30.57 ± 1.60 at.%, respectively. The O/C ratio consequently rose from 0.076 ± 0.003 to 0.424 ± 0.030 and 0.539 ± 0.053. These measurements show oxygen enrichment at the analysed surface, with the higher mean O/C ratio in the dual-beam-superimposed samples.

N/C increased from 0.030 ± 0.001 to 0.073 ± 0.007 and 0.075 ± 0.004 after single-beam and dual-beam-superimposed treatment, respectively. S/C changed less, from 0.072 ± 0.003 to 0.079 ± 0.003 and 0.081 ± 0.004. These ratios are relative to the carbon signal and do not establish absolute retention of sulfur-containing backbone structures. The higher, more variable Si/C ratio under dual-beam superposition (0.066 ± 0.029) is compatible with heterogeneous film coverage or a substrate contribution. Distinguishing these explanations requires an independent thickness or coverage measurement.

The normalised C 1s, O 1s and S 2p envelopes differ among treatments (Figure 5d–f). They provide qualitative line-shape comparisons. Quantitative chemical-state assignments would require a common binding-energy calibration and constrained component fitting. The numerical comparison here uses survey-derived atomic percentages and ratios.

The surface-composition measurements thus distinguish the representative treatments through oxygen enrichment and changes in elemental ratios. These are chemical-response criteria for evaluating the remaining film.

### 3.5. AFM Surface Morphology and Roughness

The AFM height images in Figure 3a–f reveal condition-dependent differences in the surface morphology of the PBDB-T: ITIC films. The two unetched films displayed reproducible fine-grained nanoscale topography, with film-level Rq values of 3.18 and 3.06 nm. Their group-average Rq was 3.12 ± 0.09 nm, while the corresponding Ra value was 2.39 ± 0.12 nm (Table 3). The close agreement between the two measured unetched films establishes the baseline morphology for this comparison.

After single-beam modulation, the two films exhibited Rq values of 3.70 and 10.80 nm, giving a group average of 7.25 ± 5.02 nm. The corresponding Ra value was 5.79 ± 4.09 nm. The two independently prepared films differed markedly: the rougher film displayed coarser island-like domains and deeper surface depressions, whereas the other retained a comparatively finer morphology. Thus, the representative single-beam condition was associated with a larger but highly sample-dependent topographical response. This observation should not be interpreted as direct evidence of weaker or stronger etching selectivity or molecular damage.

The dual-beam-superimposed films exhibited Rq values of 3.37 and 2.43 nm, giving a group average of 2.90 ± 0.66 nm. The corresponding Ra value was 2.29 ± 0.51 nm. These values remained comparable to those of the unetched films, and the representative height images retained a relatively fine-grained morphology without pronounced large-scale topographical roughening at the 5 μm scan scale. This morphological observation is interpreted only as a representative surface response and does not directly establish etching selectivity or lower molecular damage.

Values are presented as mean ± standard deviation from two independently prepared films per condition (n = 2). Three 5 μm × 5 μm regions were analysed for each film, and the region-level values were averaged before calculation of the group mean and standard deviation. No formal significance testing was performed because of the limited number of independent films.

### 3.6. Implications for Descriptor-Guided Process-Window Evaluation

The measured responses identify several considerations for process selection. In the 50 Pa, 180 s profilometry series, superimposed settings did not give uniformly larger apparent steps than the corresponding single-source settings. At 100 Pa and 100 s, dual-beam treatment gave higher mean O/C while retaining near-baseline mean roughness; the larger and more variable roughness response occurred after single-beam treatment. A treatment that produces a stronger chemical or morphological change therefore needs to be assessed against the acceptable surface composition, roughness and remaining thickness for the intended film application.

Comparisons are made within each measurement protocol. The optical sequence includes cumulative exposure when the source configuration changes at 50 s. XPS and AFM each contain two films per condition, and the 50 W and 50 W + 50 W settings change both source number and nominal total power. The profilometry series uses a different pressure and duration, with one local unetched-thickness reference. These factors define the scope of the observed differences and prevent their attribution solely to source number.

Window-integral SR compares the relative spectral weight in two fixed energy intervals under the stated activation-versus-damage assumption. It complements the film measurements, which establish changes in surface height, composition and morphology. The original seven-condition score order follows Equation (2), whereas Equations (3) and (4) provide a separate mean-energy reference scenario. Supplementary Section S10 expresses the window calculation as a special case of a profile-integral model and identifies the additional response-function evidence needed for molecular-selectivity prediction.

A direct test of SR screening would combine calibrated RFEA profiles with measurements on independent films treated at the same pressure, duration and geometry. Equal-total-power controls would distinguish source configuration from nominal power. Initial and residual thickness measurements on each film, together with component-resolved chemical analysis, would test the removal and molecular-selectivity interpretation. Device measurements would then determine whether the selected surface response benefits the intended application.

## 4. Conclusions

We present an RFEA-guided SR workflow for preliminary evaluation of soft-plasma conditions. Integrating the energy profile over fixed 4–6 eV and 6–8 eV windows defines a transparent spectral comparison across seven settings. The recorded scores range from 1.32 to 7.58, with the 50 + 100 label ranking first under this criterion. The mean-energy model is retained as physical context and is distinguished from the calculation used for these scores. PBDB-T:ITIC films showed treatment-dependent optical, chemical and morphological responses. At 100 Pa, dual-beam treatment gave higher mean O/C with near-baseline mean roughness, whereas single-beam treatment gave a larger and more variable roughness response. The separate 50 Pa profilometry series showed no uniform increase in apparent step height under source superposition. The workflow combines a defined spectral screening criterion with within-protocol film-response measurements for preliminary process-window evaluation.

## Figures and Tables

**Table 2:** XPS atomic percentages and atomic ratios of PBDB-T:ITIC films. The treated groups used 100 Pa for 100 s at 50 W or 50 W + 50 W. Values are the mean ± SD across two films per condition.

XPS Parameter	Unetched	Single-Beam Modulation	Dual-Beam-Superimposed Modulation
C (at.%)	83.11 ± 0.23	62.31 ± 1.63	56.84 ± 2.64
O (at.%)	6.31 ± 0.23	26.39 ± 1.15	30.57 ± 1.60
N (at.%)	2.49 ± 0.09	4.53 ± 0.29	4.23 ± 0.01
S (at.%)	6.02 ± 0.28	4.91 ± 0.08	4.63 ± 0.45
Si (at.%)	2.09 ± 0.36	1.88 ± 0.12	3.73 ± 1.49
O/C atomic ratio	0.076 ± 0.003	0.424 ± 0.030	0.539 ± 0.053
N/C atomic ratio	0.030 ± 0.001	0.073 ± 0.007	0.075 ± 0.004
S/C atomic ratio	0.072 ± 0.003	0.079 ± 0.003	0.081 ± 0.004
Si/C atomic ratio	0.025 ± 0.004	0.030 ± 0.003	0.066 ± 0.029

**Table 3:** AFM-derived surface roughness of PBDB-T: ITIC films under different plasma-modulation conditions.

AFM Parameter	Unetched	Single-Beam Modulation	Dual-Beam-Superimposed Modulation
Film-level Rq values (nm)	3.18, 3.06	3.70, 10.80	3.37, 2.43
Rq (nm), mean ± SD	3.12 ± 0.09	7.25 ± 5.02	2.90 ± 0.66
Film-level Ra values (nm)	2.47, 2.30	2.90, 8.68	2.65, 1.93
Ra (nm), mean ± SD	2.39 ± 0.12	5.79 ± 4.09	2.29 ± 0.51

## Data Availability

The data supporting the findings of this study are available from the corresponding author upon reasonable request.

## Supplementary Materials

The following supporting information can be downloaded at: https://www.mdpi.com/article/10.3390/mi17101139/s1.
