# Supplementary material for: RFEA-Derived Ion-Energy Descriptors for Preliminary Process-Window Evaluation in Dual-Beam Soft Plasma Etching of Organic Semiconductor Films
Source: Micromachines (Basel). 2026 Sep 30;17(10):1139. doi: 10.3390/mi17101139 (PMC13707040; doi:10.3390/mi17101139)
Supplement: Supplementary file 1 [file micromachines-17-01139-s001.zip › micromachines-4380139-supplementary.pdf]

Supplementary Information

RFEA-derived ion-energy descriptors for preliminary process-window evaluation in dual-beam soft plasma etching of organic semiconductor films

Rui Gao, Mengna Dai, Xin Zou *, Yuheng Wang *

Department of Medical Engineering, Second Affiliated Hospital, Army Medical University, Chongqing, 400030, China

* Corresponding author. E-mail: nano.plasma.uv@gmail.com (Yuheng Wang).

S1. AFM sample mapping and representative-image traceability

Table S1 maps the six AFM films and representative images. Treated films used 100 Pa for 100 s, at 50 W for single-beam modulation or 50 W + 50 W for dual-beam superimposed modulation (Table S12). Three 5 µm × 5 µm regions were averaged per film. Figure 5 shows one field per film and all six film-level Rq values.

Table S1. Mapping of original AFM sample identifiers to manuscript treatment labels and representative regions.

**Original sample ID**	**Treatment label in manuscript**	**Independent film**	**AFM regions used for film-level mean**	**Representative region in Fig. 5**	**Film-level Rq / Ra (nm)**
U-1	Unetched	Film 1	U-1-1, U-1-2, U-1-3	U-1-2	3.18 / 2.47
U-2	Unetched	Film 2	U-2-1, U-2-2, U-2-3	U-2-2	3.06 / 2.30
E50-1	Dual-beam superimposed modulation	Film 1	E50-1-1, E50-1-2, E50-1-3	E50-1-2	3.37 / 2.65
E50-2	Dual-beam superimposed modulation	Film 2	E50-2-1, E50-2-2, E50-2-3	E50-2-2	2.43 / 1.93
E50-2-1	Single-beam modulation	Film 1	E50-2-1-1, E50-2-1-2, E50-2-1-3	E50-2-1-1	3.70 / 2.90
E50-2-2	Single-beam modulation	Film 2	E50-2-2-1, E50-2-2-2, E50-2-2-3	E50-2-2-2	10.80 / 8.68

Film IDs and region IDs must be read together with the treatment and parent-film columns. E50-2-1 and E50-2-2 are independent single-beam films; E50-1 and E50-2 are independent dual-beam films. Similarly named regions under a different parent film are distinct records. The original height fields and colour scales are retained. Group summaries retain the reported precision in the main manuscript because displayed film-level values are rounded.

AFM used a Bruker Dimension Icon. Three region-level Rq and Ra values were averaged per film before calculating the mean and sample SD across two films per treatment. The 18 regions represent six films; Table S1 maps their representative fields to Figure 5.

S2. MATLAB data and the SR summary

DATA.zip contains 14 MATLAB files: seven nmatrix arrays in data/density and seven corresponding Vmatrix arrays in data/voltage. Each array has 1001 rows and 41 columns, without embedded axis names or units. The separate Density_summary.csv contains the seven SR scores from the window-integral processing described in Section S12. Those scores were read directly for plotting. Table S2 preserves the original filenames and numerical fields. No separate variable named SR is present in the recovered matrix files.

For the archived mean and signed-width fields, x and y denote the first and second matrix columns in their stored row order. The mean is the sum of their pointwise products divided by the sum of y. The signed width is the last x value minus the first x value among rows for which y reaches at least half its maximum. First and last refer to row order, not to the smallest and largest coordinate values. These operations reproduce the seven archived mean and signed-width entries to relative tolerance 2×10^{-14}. They describe the arithmetic of those two fields and are not the window-integral SR calculation.

In 50meV-20Vn.mat, 100meV-20Vn.mat and 150meV-20Vn.mat, the first-column span is about 3.4–3.9×10^{-14} of its mean. Two other records yield negative widths in stored row order. These arithmetic fields are retained without interpreting them as calibrated mean ion energies or physical FWHMs. The operational SR uses areas on the processed energy axis, as defined in main-text Eq. (2); it is not calculated from the mean or signed-width columns. All 41 matrix columns are preserved.

Table S2. Original numerical summary. The mean and signed-width columns retain their archived arithmetic values with unassigned physical units. The SR column contains the dimensionless window-integral scores read from Density_summary.csv. Short condition labels elsewhere in the text refer to these filenames.

**Archived nmatrix filename**	**Mean arithmetic**	**Signed-width arithmetic**	**Window SR**
50meV-20Vn.mat	1.121736e+09	+3.838539e-05	1.5345024
100meV-20Vn.mat	7.761175e+08	+2.682209e-05	1.4376942
150meV-20Vn.mat	1.225865e+09	+4.720688e-05	1.3198106
50+50meV-20Vn.mat	2.408183e+09	+3.602109e+09	5.1224195
100-50meV-20Vn.mat	2.071367e+09	-4.063440e+09	4.0232599
150-50meV-20Vn.mat	3.797947e+09	-1.355457e+10	3.5724647
50+100meV-20Vn.mat	4.031335e+09	+1.338230e+10	7.5809155

S3. Current-transient files and late-window averages

The earlier record describes seven current-transient CSV files in two folders in DATA.zip. Folder A is DATA/CSV. Folder B has a one-character Chinese name (Unicode U+738B, romanised as Wang). TIME1 is the recorded time in seconds and CURR1 is current in amperes. For every file, the late-window mean uses all samples satisfying $t > 0.30t\_max$, without interpolation, smoothing, baseline subtraction or status-code filtering. Folder A files retain 42 of 60 rows; folder B files retain 21 of 30. The folder labels in Table S3 distinguish files with identical basenames. These current-versus-time records are distinct from the collector-current/retarding-potential inputs in Eq. (S6) and the I–V scans in Figure 3e.

Table S3. Late-window current means for all seven recovered records, with folders A and B defined above. Nominal bias and duration are taken from each record.

**Record**	**Nominal bias (V)**	**Retained / total**	**Duration (s)**	**Mean I (μA)**
A/100mv.csv	0.10	42 / 60	118	3.608093
A/100mv_1.csv	0.10	42 / 60	59	3.568840
A/200mv.csv	0.20	42 / 60	177	0.013447
A/300mv.csv	0.30	42 / 60	177	0.018978
B/100mv.csv	0.10	21 / 30	29	3.434408
B/200mv.csv	0.20	21 / 30	29	3.569187
B/300mv.csv	0.30	21 / 30	29	3.504576

S4. Consistency check of the paired MATLAB matrices

In the earlier paired-matrix check, each nmatrix array was integrated twice by cumulative trapezoidal integration along its row axis, with unit index spacing and zero initial integrals. A single least-squares coefficient, $-2.0661675205687634 \times 10^{-30}$, fitted across all seven pairs maps these double integrals to Vmatrix. The relative Frobenius-norm errors are 0.0031–0.0253% (Table S4). This close numerical relation is consistent with a density–potential calculation such as the Poisson relation discussed in the earlier SI [16], Note S2, Eq. (7).

Table S4. Paired-matrix numerical check using one shared coefficient. $\mathrm{Error}(\%) = 100 \times \frac{\|\mathbf{V} - cD_2(\mathbf{N})\|_\mathrm{F}}{\|\mathbf{V}\|_\mathrm{F}}$, where D_2 denotes the stated double index integration. Matching Vmatrix filenames replace the terminal Vn with VV.

**nmatrix filename**	**Corresponding Vmatrix filename**	**Relative error (%)**
50meV-20Vn.mat	50meV-20VV.mat	0.017150
100meV-20Vn.mat	100meV-20VV.mat	0.006241

nmatrix filename	Corresponding Vmatrix filename	Relative error (%)
150meV-20Vn.mat	150meV-20VV.mat	0.003091
50+50meV-20Vn.mat	50+50meV-20VV.mat	0.025250
100-50meV-20Vn.mat	100-50meV-20VV.mat	0.012090
150-50meV-20Vn.mat	150-50meV-20VV.mat	0.004359
50+100meV-20Vn.mat	50+100meV-20VV.mat	0.012295

S5. Analytical reference curves and prior-model context

The source of the physical relation is Ref. [16], published SI, Note S3, Eq. (13). The main article points to Eq. (S12), whereas the SI itself numbers the relation (13). With an activation-energy difference of 6.4 − 2.8 = 3.6 eV, the following explicit positive-width branch reproduces the separate reference-curve file:

$$f(E;w)=\exp\left[\frac{3.6\text{eV}}{E+\dfrac{w}{2\times 2.355}}\right],\qquad E_k=\left(5+\frac{10k}{49}\right)\text{eV},\qquad k=0,\ldots,49 \tag{S1}$$

Equation (S1) fixes the otherwise unspecified proportionality factor to one and chooses the positive width branch for the reference calculation. These choices reproduce the analytical reference file. The seven condition scores instead use the window-integral definition in main-text Eq. (2). The source model assigns activation energies of 2.8 and 6.4 eV. An arbitrary matrix coordinate cannot be substituted for energy without a documented conversion. The mean-energy reference formulation uses the mean energy without the positive-width offset. The branch in Eq. (S1) is retained only to reproduce this specific analytical file.

Ref. [16] reports a CNN–LSTM spectral-prediction model separately from the physical relation examined here. No machine-learning training or inference was performed in the present study. The prior relation supplies physical context; the numerical 4–6 eV and 6–8 eV integration windows are the operational definition adopted in this study, rather than a formula quoted from that reference.

The published SI reports five-batch outcomes in Table S5, including tabulated etch depths of 7.9–8.3 nm and mobilities of 1.08–1.15 $cm^2\ V^{-1}\ s^{-1}$. The source supplies model context and prior process results, rather than the component-resolved validation needed for a condition-specific molecular-selectivity interpretation.

Table S5. Representative rows from the recovered 50-point reference calculation. Row numbers are one-based. The energy coordinates are retained as recorded, without assigning values to nominally rounded energies. Lower and upper values are deterministic width-sensitivity limits. Displayed rows are 1, 11, 21, 31, 41 and 50.

**Energy (eV)**	**Reference ratio**	**Lower limit**	**Upper limit**
5.000000	1.884889	1.871685	1.898490
7.040816	1.594098	1.588027	1.600293
9.081633	1.446018	1.442564	1.449525
11.122449	1.356675	1.354455	1.358922
13.163265	1.297015	1.295471	1.298575
15.000000	1.258097	1.256929	1.259276

The recovered ion_energy_distribution.csv contains two analytical Gaussian profiles centred at 10 eV, with FWHM values of 3.2 and 2.5 eV. Both are reproduced by $\exp[-4 \ln(2)((E - 10)/w)^2]$, with maximum absolute differences below 8×10^{-15}. Figure S1a shows all supplied rows as model profiles. In sensitivity_ratio.csv, all 50 rows are reproduced by Eq. (S1): $w = 3.2$ eV for the central curve, 3.5 eV for the lower curve and 2.9 eV for the upper curve, with maximum absolute differences below 7×10^{-15}. The envelope in Figure S1b is deterministic width sensitivity, not a confidence interval. The central ratio decreases across the provided 5–15 eV range; these curves alone do not identify a 9–11 eV optimum.

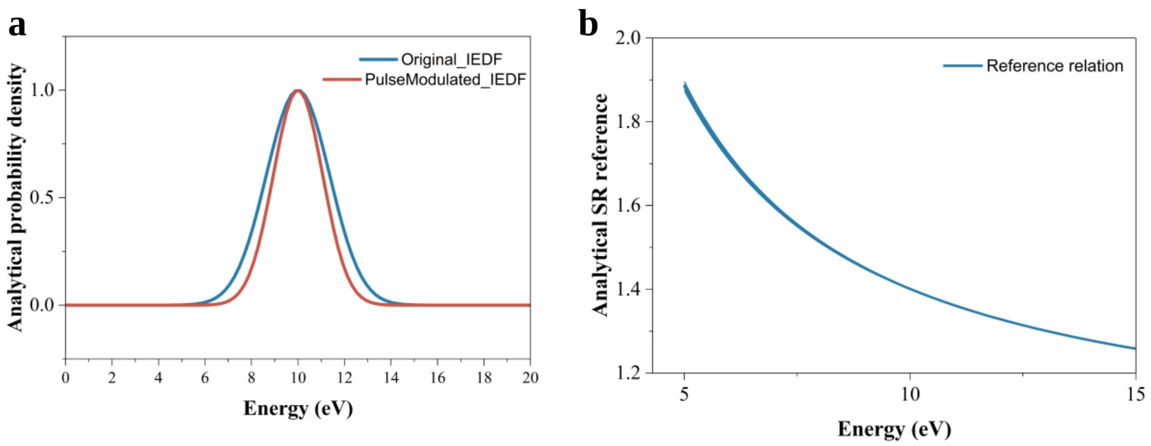

Figure S1. Reconstructed analytical reference calculations. (a) All supplied rows of two peak-normalised Gaussian profiles centred at 10 eV, with FWHM values of 3.2 and 2.5 eV. (b) All 50 rows of the response relation evaluated with Eq. (S1). The line uses w = 3.2 eV and the envelope uses w = 2.9–3.5 eV. The profiles are analytical examples, and the envelope shows deterministic parameter sensitivity without a statistical confidence level. These calculations are separate from the experimental film measurements and historical condition scores.

S6. Data and analysis-file availability

This SI supports the RFEA-guided SR screening objective of the main manuscript. Section S12 specifies the two-window calculation, its ranking rule and its distinction from the mean-energy reference model. Section S10 provides the general profile-integral formulation. Sections S1 and S7–S9 document the complementary film measurements. Sections S2–S5 and S11 retain the numerical records, analytical examples and comparison with the reference relation.

The available records include DATA.zip with the 14 MATLAB matrices, seven separately identified current-transient files and analytical reference files, together with the separate Density_summary.csv score summary. The archived matrices and CSV are retained as supplied. The filenames iteration_convergence.csv and grid_convergence.csv are legacy references and do not denote a new machine-learning analysis. The separate RFEAraw_and_calibration teaching package labels T01–T07 as SYNTHETIC_TEACHING_NOT_EXPERIMENTAL; these examples are not used as experimental inputs or as the source of the seven reported SR scores.

The film records described here include AFM height images and roughness summaries for six films (Table S1), and 42 unique profilometry scans, with three scans per numbered specimen (Section S7). Duplicate exports are counted once. The displayed profiles and numerical summaries support the within-protocol comparisons in the main manuscript; native acquisition records fall under its Data Availability Statement.

References cited in this Supplementary Information follow the numbering of the main manuscript.

S7. Stylus profilometry across etched and unetched film regions

S7.1. Sample mapping and common analysis definition

The numbered specimens belong only to the profilometry series and each has three CSV scans. Specimens 1–13 were treated at 50 Pa for 180 s. Specimen 14 was designated for measurement of unetched film thickness. The power-component order is retained: 50 W + 100 W and 100 W + 50 W are distinct settings. The request specifies three scans across etched and unetched film regions, and the authors confirm this geometry for specimens 1–13. Their exact scan positions are not encoded in the CSVs.

Table S6. Confirmed profilometry specimen-to-condition mapping.

Specimen IDs	Power setting	Mode	Pressure (Pa) / time (s)	Scans
1, 2	50 W	Single-source	50 / 180	6
3, 4	100 W	Single-source	50 / 180	6
5, 6	150 W	Single-source	50 / 180	6
7, 8	50 W + 50 W	Superimposed	50 / 180	6
9, 10	100 W + 50 W	Superimposed	50 / 180	6
11, 12	150 W + 50 W	Superimposed	50 / 180	6
13	50 W + 100 W	Superimposed	50 / 180	3
14	Unetched	Thickness reference	Not etched	3

Each scan contains 5401 points over 899.6668 µm. Export settings are a nominal 900 µm length, 18 s duration, 0.166605 µm sampling, 2 µm stylus radius and 3 mg force. Heights in Å were divided by 10 to obtain nm. Instrument levelling and offsets were retained; no smoothing or removal of recorded points was applied. The common step analysis uses window medians directly. The separate scratch calculation in Section S7.4 uses an interpolated local film baseline.

For every scan, the primary analysis uses the median height in L = 50–150 µm and R = 700–800 µm. Each primary window contains 600 recorded points. The signed height difference is defined below.

$$\Delta h = \text{median}[h(x), x \text{ in } R] - \text{median}[h(x), x \text{ in } L]$$

The windows lie outside the main transitions, although some include a residual slope. For specimens 1–13, the documented etched/unetched boundaries and positive Δh values support interpreting these steps as apparent surface recession. Specimen 14 has a different measurement purpose. Its common-window values are retained only as diagnostics and are neither interpreted as thickness nor subtracted from treated steps. Tables S9 and S10 assess alternative windows.

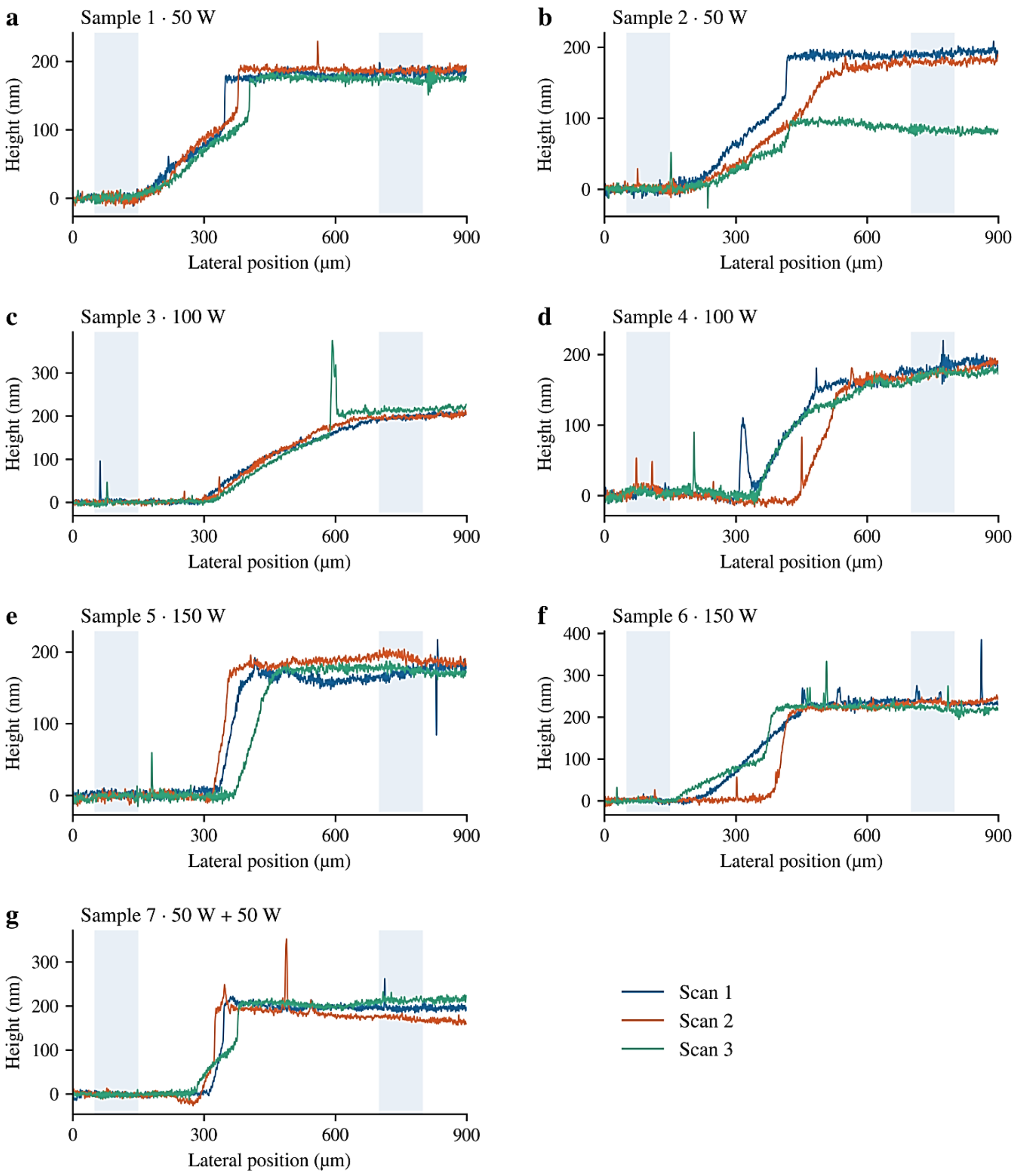

Figure S2. Complete exported profilometry scans for specimens 1–7. Panels (a–g) correspond to specimen IDs 1–7 in order. Each panel contains all three scans, with the original vertical offsets and all recorded points retained. Shading marks the primary left and right analysis windows at 50–150 and 700–800 μm. Height axes use panel-specific ranges so that the recorded excursions remain visible. Conditions are given in Table S6. All scans are retained and analysed with the same window definitions.

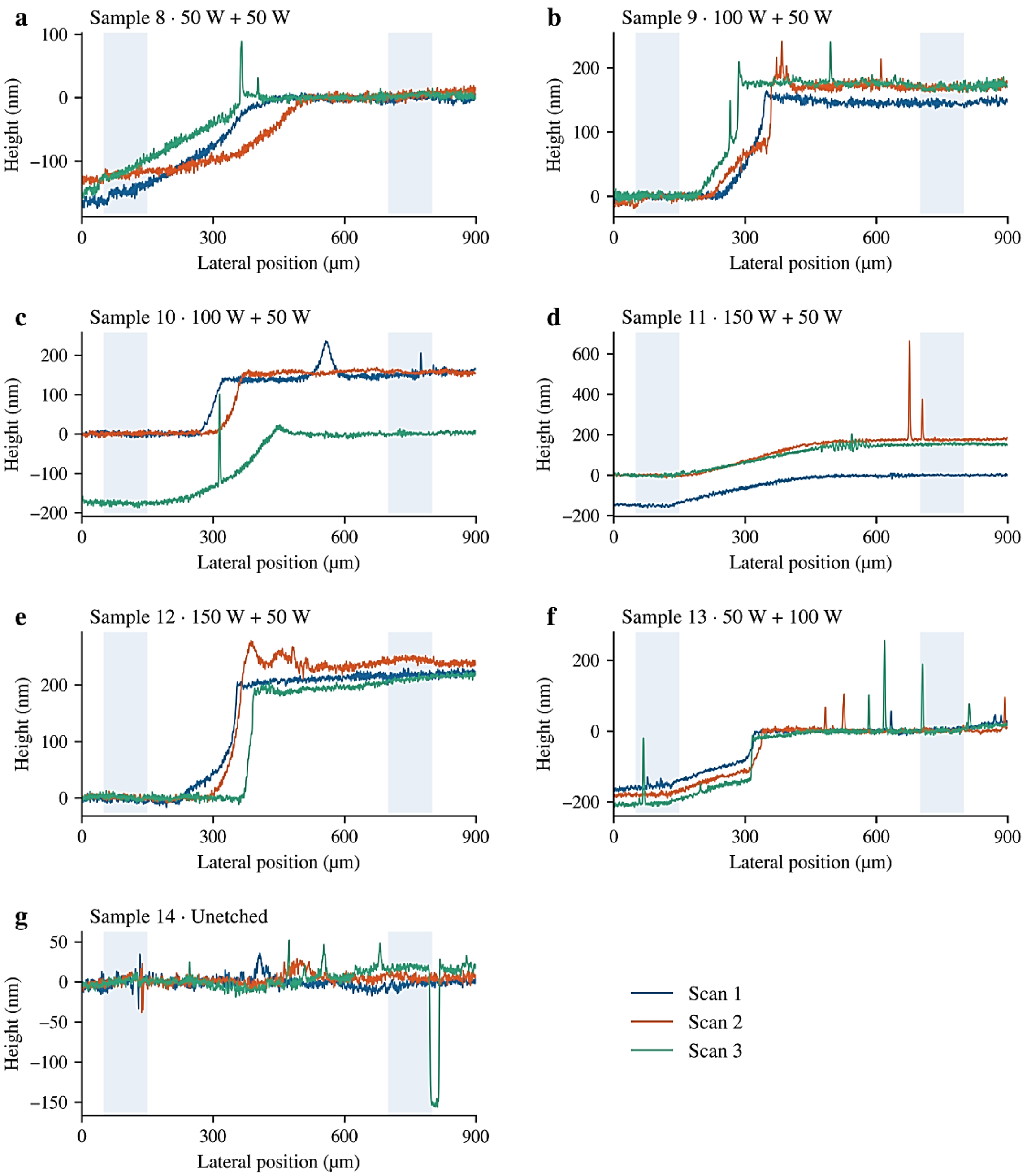

Figure S3. Complete exported profiles for specimens 8–14. Panels (a–g) correspond to IDs 8–14 in order. Plotting conventions follow Figure S2. Original vertical offsets, slopes and local excursions are retained. Specimens 8–13 were scanned across etched and unetched film regions. Specimen 14 in panel (g) is the unetched-film thickness reference. The authors identify the trough near 796–816 μm in scan 14-3 as a scratch reaching bare glass. Its local thickness analysis is shown in Figure 2b and described in Section S7.4. The shaded common windows in panel (g) are diagnostic windows and do not measure this thickness. All three scans remain available for inspection.

S7.2. Scan-level values and specimen-level summaries

Table S7. Primary-window height differences for all scans. All height values are in nm. The specimen mean and SD are calculated from its three scan values. For specimen 14, these are diagnostic window differences, not measured film thickness or a zero-etch control.

ID	Power setting	Scan 1	Scan 2	Scan 3	Mean	SD of scans
1	50 W	182.7	187.5	172.3	180.8	7.8
2	50 W	192.0	177.0	82.9	150.6	59.1
3	100 W	193.6	196.5	213.7	201.3	10.8
4	100 W	171.6	164.9	169.1	168.5	3.4
5	150 W	169.4	195.6	177.5	180.8	13.4
6	150 W	236.3	233.6	222.7	230.9	7.2
7	50 W + 50 W	195.0	174.6	214.8	194.8	20.1
8	50 W + 50 W	148.4	124.6	119.3	130.8	15.5
9	100 W + 50 W	144.4	169.2	167.7	160.4	13.9
10	100 W + 50 W	151.4	155.6	176.0	161.0	13.1
11	150 W + 50 W	149.2	175.3	155.8	160.1	13.6
12	150 W + 50 W	220.3	244.3	208.6	224.4	18.2
13	50 W + 100 W	160.2	179.3	203.5	181.0	21.7
14	Unetched	-3.8	3.5	16.7	5.5	10.4

The three scans are repeated measurements of one numbered specimen and are not counted as three independent treatment replicates. No scan was excluded. The between-scan SD reflects spatial and measurement variation within that specimen.

Table S8. Condition-level summary of etched-to-unetched step heights for specimens 1–13. Group means and SDs use specimen means. The n column counts specimens. All values are in nm. Specimen 14 is assessed separately in Section S7.4.

Power setting	Specimen IDs	n	Mean Δh	SD across specimens
50 W	1, 2	2	165.7	21.3
100 W	3, 4	2	184.9	23.2
150 W	5, 6	2	205.9	35.4
50 W + 50 W	7, 8	2	162.8	45.3
100 W + 50 W	9, 10	2	160.7	0.4
150 W + 50 W	11, 12	2	192.3	45.5
50 W + 100 W	13	1	181.0	Not estimable

The 50 W + 100 W condition has one specimen. Its scan SD is 21.7 nm and is not a between-specimen error bar. The small between-specimen SD for 100 W + 50 W reflects similar specimen means, not equally small scan-level variation. For specimen 14, the diagnostic scan SD of 10.4 nm does not estimate uncertainty in the initial film thickness.

S7.3. Sensitivity to analysis-window placement and interpretation

The same primary and alternative window definitions were applied to all 42 scans. For each pair, Δh was recomputed from window medians and then averaged over the three scans of each specimen. This analysis examines placement sensitivity without selecting different windows for different power settings.

Table S9. Fixed lateral-window pairs used in the sensitivity analysis.

Window pair	Left window (μm)	Right window (μm)
A (primary)	50–150	700–800
B	20–120	650–750
C	80–180	750–850

Table S10. Specimen-mean height differences under each window pair, in nm. Range is the maximum minus the minimum of the three specimen means. It is not a confidence interval or total measurement uncertainty.

ID	Power setting	A	B	C	Range
1	50 W	180.8	180.4	178.9	1.9
2	50 W	150.6	150.8	151.2	0.5
3	100 W	201.3	199.6	204.3	4.7
4	100 W	168.5	163.8	173.9	10.1
5	150 W	180.8	179.7	179.2	1.6
6	150 W	230.9	230.7	227.0	3.9
7	50 W + 50 W	194.8	194.0	193.6	1.2
8	50 W + 50 W	130.8	134.7	124.8	9.9
9	100 W + 50 W	160.4	162.3	161.3	1.8
10	100 W + 50 W	161.0	160.8	162.9	2.1
11	150 W + 50 W	160.1	159.0	158.0	2.1
12	150 W + 50 W	224.4	219.9	225.4	5.5
13	50 W + 100 W	181.0	181.6	178.4	3.2
14	Unetched	5.5	5.8	4.4	1.5

Specimen-mean ranges across window choices were 0.5–10.1 nm. Slopes in sample 8 and variation in sample 4 remain visible. Window medians limit isolated excursions but do not correct tilt. The measurement request and author confirmation establish the etched/unetched boundary.

The 50 Pa, 180 s series is separate from the 100 Pa, 100 s XPS/AFM comparisons, the optical sequence and the MATLAB archive. Its steps represent apparent surface recession. Section S7.4 establishes one local

unetched-film thickness, but the initial thickness of each treated specimen and its residual film state remain unknown. No thickness normalisation, SR–depth regression or etch-rate conversion is reported. The steps do not quantify molecular selectivity.

S7.4. Unetched-film thickness reference

Specimen 14 is the unetched-film thickness reference. The authors confirm that the trough near 796–816 μm in scan 14-3 is an intentional scratch reaching bare glass. Film shoulders were sampled at 775–790 and 825–840 μm, with 90 points in each window. Their median heights were 16.8317 and 15.5969 nm at median lateral positions of 782.4602 and 832.4417 μm. A straight local film baseline was interpolated between these two positions. The scratch-floor window at 800–812 μm contains 72 points. Local thickness was calculated as the median of the baseline height minus the recorded floor height at these points, giving 168.6348 nm, reported as 168.6 nm.

Shifting all three local windows together by −2 and +2 μm gave thicknesses of 169.7467 and 169.1595 nm. The three window choices therefore span 168.6–169.7 nm. This is a placement-sensitivity check, not a confidence interval. Only one scratch location has a confirmed film-to-glass assignment. Scans 14-1 and 14-2 are retained, but are not counted as thickness replicates.

The common windows used elsewhere, 50–150 and 700–800 μm, yield diagnostic differences of −3.8, 3.5 and 16.7 nm for the three scans of specimen 14. Their mean of 5.5 nm is not a thickness measurement. The instrument screenshots also show Total_ASH values of 0.67, 0.99 and −3.61 nm at other cursors, rather than across the confirmed scratch. These values are not subtracted from treated-specimen steps. Because several treated steps exceed the local 168.6 nm reference, starting thickness and remaining film must be assessed on the corresponding specimens before calculating removal fractions.

S8. Supporting records for profilometry and controller settings

The profilometry request submitted through Kexue Zhinanzhen (order 26082270227, page 2) specifies three scans across the unetched/etched film boundary and asks that both regions be covered. This agrees with the geometry confirmed for specimens 1–13. Specimen 14 was instead designated for unetched film-thickness measurement. The request is a measurement instruction, not a thickness-calibration certificate or a record of the masking procedure. The sample dimensions, 20 mm × 20 mm × 1 mm, refer to the submitted specimen as a whole.

Table S11. Values transcribed from the controller photograph. Translations describe the displayed fields; values and unit labels are retained exactly.

Displayed field (English translation)	Value	Displayed unit
Cycle time setting	30000	s
RF power supply delay setting	10	s
Balance valve delay setting	30	s

The photograph is a controller-state record supplied during revision. It does not establish the settings used during each earlier experiment. The 30000 entry is preserved with the displayed s label; no undocumented scaling or

conversion is applied. The two delay fields are not RF oscillation periods. Frequency, waveform, duty cycle and relative phase cannot be recovered from this page, and the delays are not subtracted from the reported 180 s treatment time.

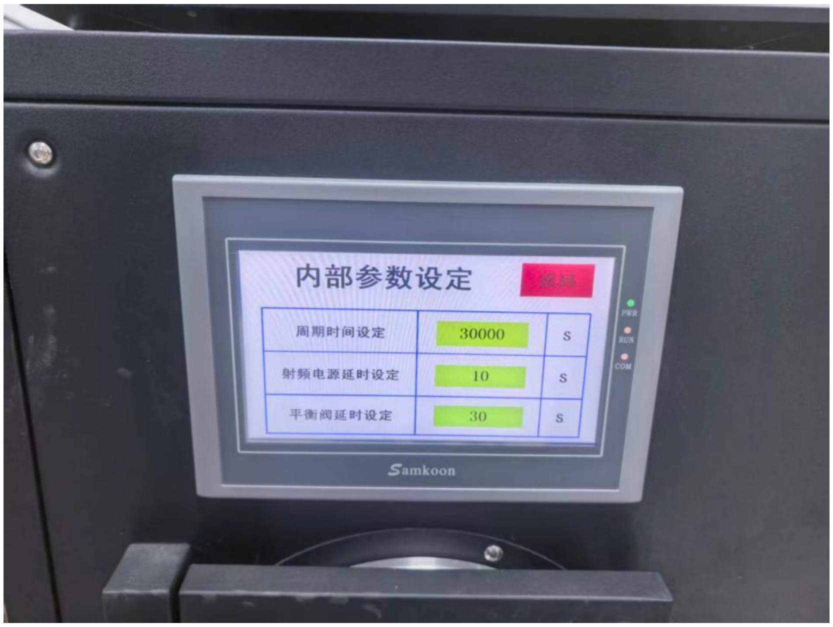

Figure S4. Original photograph of the internal-parameter page. From top to bottom, the rows show cycle time, RF power supply delay and balance valve delay. The Samkoon name identifies the visible operator-interface hardware; it is not used here as the plasma-source manufacturer or model.

S9. Treatment conditions and optical and electrical measurements

XPS and AFM were performed by external testing services on separately prepared films, with two films per group for each method (Table S12). The 50 W and 50 W + 50 W settings change both the number of active emitters and the sum of their nominal powers. The comparison does not isolate a source-number effect. The pressure and duration also differ from those of the 50 Pa, 180 s profilometry series, so the methods do not provide matched measurements of one common set of treatment conditions.

XPS used a Thermo Scientific K-Alpha instrument without Ar^+ sputter cleaning. Survey atomic percentages used the instrument sensitivity factors, whereas background-subtracted and independently normalised high-resolution spectra were used for qualitative line-shape comparison. AFM used the protocol in Section S1. The XPS and AFM film sets were separately prepared under the same endpoint conditions, so their association is interpreted at the treatment-group level.

Table S12. Confirmed treatment conditions for the XPS and AFM endpoint comparisons. Duration is the stated treatment time for each group. The ordered power components are retained as nominal settings.

Group	Pressure (Pa)	Nominal power (W)	Duration (s)
Unetched	Not treated	Not treated	0
Single-beam modulation	100	50	100
Dual-beam superimposed modulation	100	50 + 50	100

The spectroscopic apparatus was developed at Xi'an Jiaotong University. A xenon lamp illuminated the films in a darkened environment. Absorbance and reflection spectra were acquired during etching at 50 Pa and a nominal setting of 50 W, over a 100 s protocol with 1 s sampling (Table S13). Figure 3a,b presents the early interval, and Figure 3f covers 100 s. The latter is labelled 0–50 s for one-emitter operation and 50–100 s for simultaneous two-emitter operation. The second interval begins after 50 s of prior exposure on the same film. The nominal 50 W record does not resolve the individual source powers during simultaneous operation. The map reports elapsed time because no independent time-to-depth calibration is available. Detector integration time and the detailed reference-correction procedure were not documented in the available records.

Table S13. Optical acquisition conditions during plasma etching. The recorded nominal setting is 50 W; the individual source powers in the two-emitter interval are not specified by this entry. The sampling interval denotes acquisition cadence.

Measurement	Pressure (Pa)	Nominal power (W)	Treatment (s)	Sampling interval (s)
Absorbance	50	50	100	1
Reflection map	50	50	100	1

Absorbance is expressed as $A = -\log_{10}(I/I_0)$, where I and I_0 denote sample and reference intensities at the corresponding wavelength. Figure 3b presents spectra at 0, 8, 16, 25, 33 and 42 s. The frequency-response measurements in Figure 3c,d used the unetched film at nominal DC biases of 0 and 0.2 V. Sequential I–V scans of the same unetched film covered −1.0 to 0.9 V; baseline-drift correction preceded the pointwise median and interquartile-range summary in Figure 3e. These electrical measurements describe the baseline and technical repeatability.

S9.1. Electrical measurement apparatus

Baseline impedance measurements used a TH2840B precision LCR meter (Tonghui). Probe contacts were positioned under microscope observation using PM-004A probe holders (MyProber (Guangzhou) Technology Co., Ltd.). The study measured 10^3–10^5 Hz at nominal DC biases of 0 and 0.2 V.

(a)

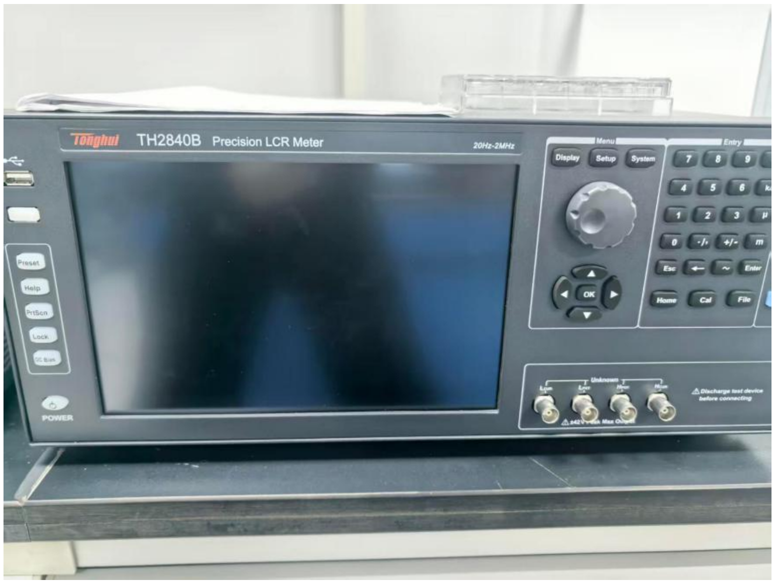

(b) **(c)**

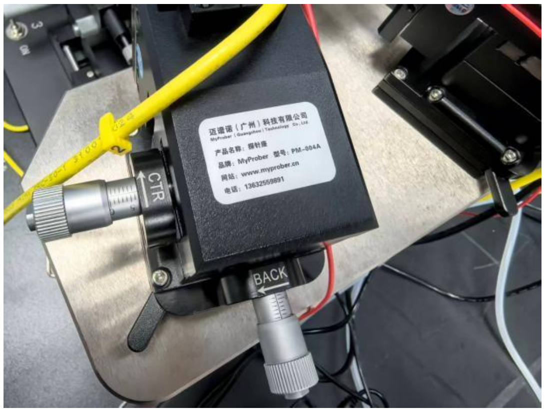

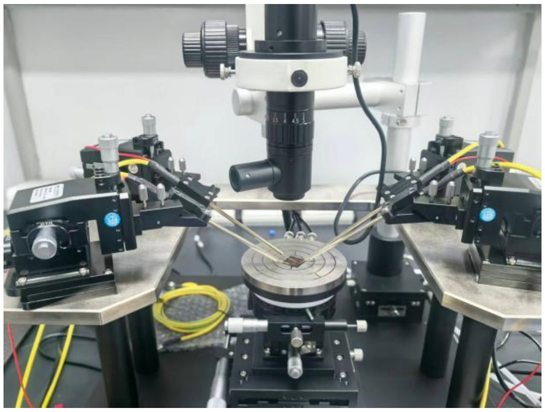

Figure S5. Electrical measurement apparatus. (a) Tonghui TH2840B precision LCR meter. (b) Identification label of a MyProber PM-004A probe holder. (c) Microscope-assisted probing arrangement. The 20 Hz–2 MHz marking in panel (a) is the instrument range; the study frequency interval is specified above.

S10. Profile-integral SR formulation and response assumptions

A ratio of profile-weighted contributions provides a common mathematical description of the window index and more general response models. In the operational calculation, the weights select the fixed 4–6 eV and 6–8 eV intervals. Replacing these interval weights with independently supported reaction-yield functions would extend the model to energy-dependent material responses. The mean-energy reference approximation in main-text Eqs. (3)–(4) is retained separately because evaluating a response at the mean does not generally equal integrating it across a profile.

S10.1. Definition and physical inputs

Let $g_i(E)$ be a nonnegative, flux-weighted ion-energy profile for condition i at the sample position. Its energy axis and instrument response must be documented. Define the normalised profile $p_i(E)$ over the full measured energy range Ω.

$$p_i(E) = \frac{g_i(E)}{\int_\Omega g_i(E')\mathrm{d}E'} \tag{S2}$$

The normalising integral must be finite and positive. With E in eV, $p_i(E)$ has units of eV^{-1} and integrates to one. Multiplying $g_i(E)$ by a positive constant leaves the normalised profile unchanged, so Eq. (S2) separates profile shape from total incident flux.

Let $K_a(E)$ and $K_\beta(E)$ denote two nonnegative weighting functions. For the operational window index, K_a equals one in 4–6 eV and zero outside that interval, and K_β equals one in 6–8 eV and zero outside that interval. In a more general physical model, these weights could instead represent alkyl-scission and backbone-damage yields per incident ion under a commensurate response convention. The two profile-weighted contributions are defined below.

$$A_i = \int_\Omega p_i(E)K_\mathrm{a}(E)\mathrm{d}E, \qquad B_i = \int_\Omega p_i(E)K_\mathrm{b}(E)\mathrm{d}E \tag{S3}$$

$$\mathrm{SR}_{\mathrm{model},i} = \frac{A_i}{B_i}, \qquad B_i > B_{\min} \tag{S4}$$

With the interval weights above, A_i and B_i are the fractions of the normalised profile contained in the lower and upper windows. Equation (S4) then reduces to main-text Eq. (2), because the common profile-normalisation area cancels. Bmin denotes a declared denominator-resolution threshold for numerical evaluation; it is not an additional fitted parameter used to alter the recorded scores. A denominator at or below the applicable resolution is unresolved. A_i and B_i should accompany a recomputed score because a large ratio may arise from a very small denominator.

A common multiplicative intensity factor cancels in the window ratio, but an energy-dependent transmission correction generally does not. Comparable energy referencing and analyser-response treatment are therefore required across conditions [55]. For a physical reaction-yield interpretation, a fixed effective ion population and a comparable surface state are additional assumptions. The ratio alone does not determine total ion dose or absolute reaction rates.

The interval weights are an operational screening model. Interpreting the lower window as favourable alkyl activation and the upper window as competing backbone damage is a phenomenological assignment. The windows are not chemically exclusive channels or independently measured ion-impact thresholds. Visible absorbance, survey O/C, AFM roughness and total step height do not separately determine reaction-yield functions. Accordingly, the reported SR is a spectral screening index; a molecular etch-selectivity ratio would require independent channel-specific response information.

S10.2. Source operation and exposure weighting

For two emitters operating simultaneously, use the energy profile measured with both emitters active at the sample position. Adding separately measured single-emitter profiles is an additional approximation and is not assumed here. A 50 W + 50 W setting is not converted to an ion energy. If the profile changes during treatment, interval profiles must be weighted by their incident ion fluences, with response kernels held fixed only if that approximation is justified.

$$\overline{p}(E) = \frac{\sum_k \Phi_k p_k(E)}{\sum_k \Phi_k} \tag{S5}$$

Here Φ_k is the ion fluence during interval k. Optical spectra acquired every 1 s do not supply Φ_k or establish constant ion flux. The separate reference function sr_from_exposure.m is specified to implement this possible fluence-weighted extension; it is not identified as the historical seven-score processing script. Without fluence information, equal weighting is only a stated scenario, not a measured exposure average.

S10.3. Optional response family and sensitivity analysis

One possible phenomenological kernel family for a sensitivity study is $K_j(E) = \begin{cases} a_j \left(1 - \frac{E_{th,j}}{E}\right)^{p_j}, & E > E_{th,j}, \\ 0, & E \le E_{th,j}. \end{cases}$ and zero otherwise, with nonnegative a_j and $E_{th,j}$ and positive p_j. This is a proposed parameterisation, not an established law for PBDB-T:ITIC. No numerical defaults are assigned. Gas-phase bond energies are not substituted for ion-impact thresholds. Parameters should be supported independently and held consistent across conditions, with any change justified by different physical inputs.

Varying response-function parameters or window boundaries gives a sensitivity range, not a statistical confidence interval. The seven reported scores use the same fixed windows. A claim that their order remains unchanged under alternative windows would require an additional calculation. No such robustness claim is inferred from the width-sensitivity curves in Section S5.

S10.4. Implementation and verification boundary

The separate reference MATLAB implementation is specified to evaluate the general Eq. (S4) with trapz(E,y), retaining nonuniform energy spacing. It requires finite, nonnegative data, strictly increasing energy axes and weight coverage of the profile range. It does not silently sort, smooth, clip or extrapolate inputs. This implementation describes an independent numerical specification, not the recovered source of the historical SR column. Section S12.3 gives the boundary handling required to reimplement the fixed-window definition.

The earlier implementation record describes input templates, an optional weight generator, an exposure-weighting function and nine mathematical self-tests, with corresponding checks in an independent Python implementation. It separately reports ten descriptor-model tests, as summarised in Section S12. MATLAB and Octave execution was not included in that record. These tests concern the separate reference implementations and artificial fixtures. They do not constitute an independent recalculation of the seven archived window scores.

S11. Distinction between window scores and the exponential reference model

The following diagnostic retains the numerical comparison of Table S2 with the positive-width exponential relation in Eq. (S1). Its purpose is to show why the reference model and the operational window index must not be treated as interchangeable definitions. Define $q_i = \mu_i + |w_i|/(2 \times 2.355)$, using the archived arithmetic fields solely for this diagnostic. Taking $|w_i|$ supplies a nonnegative width in the comparison; it does not turn that field into a physical FWHM.

Under an increasing affine coordinate map, the effective denominator would be $a\, q_i + b$, with $a > 0$. For a positive denominator, a shared positive prefactor C and the reference activation-energy difference of 3.6 eV, $C \exp[3.6/(a\, q_i + b)]$ must decrease as q_i increases. However, the 50meV-20Vn row has both a larger q and a larger recorded SR than the 100meV-20Vn row (Table S14). This excludes that exponential conversion as a reconstruction of the stored scores. It does not contradict their window-integral definition, which depends on the two spectral areas rather than q.

For the diagnostic scenario C = 1 and b = 0, each stored score would imply $a_i = 3.6/[q_i \ln(SR_i)]$. These forced scales differ by a factor of 49.4. They are algebraic consequences of imposing the exponential model on a different index, not measured calibration coefficients. They are retained for comparison and are not applied to the data.

Table S14. Diagnostic comparison of the recorded window SR with the exponential reference relation. File labels omit the .mat suffix. q retains unassigned matrix units. Forced a_i is an algebraic diagnostic and is not used for energy calibration or SR calculation.

File label	Archived SR	q	Forced a_i
50meV-20Vn	1.5345024	1.121736e+09	7.494782e-09
100meV-20Vn	1.4376942	7.761175e+08	1.277673e-08
150meV-20Vn	1.3198106	1.225865e+09	1.058316e-08

File label	Archived SR	q	Forced a_i
50+50meV-20Vn	5.1224195	3.172962e+09	6.945200e-10
100-50meV-20Vn	4.0232599	2.934093e+09	8.813747e-10
150-50meV-20Vn	3.5724647	6.675776e+09	4.235309e-10
50+100meV-20Vn	7.5809155	6.872588e+09	2.585957e-10

The numerical reconstruction reproduces all seven archived mean and signed-width entries. Separately, all 50 rows of the central, lower and upper analytical reference curves are reproduced with maximum absolute errors below 7×10^{-15} in Python. These checks concern the archive-field arithmetic and Eq. (S1), respectively. Neither check is a calculation of the two-window SR values. The reported seven scores remain the unchanged values in Density_summary.csv.

The descending SR order is 50+100 (7.5809155), 50+50 (5.1224195), 100−50 (4.0232599), 150−50 (3.5724647), 50 (1.5345024), 100 (1.4376942) and 150 (1.3198106). These shortened labels identify Table S2 rows and retain the original filename notation. They do not independently define an energy calibration or a mapping to the profilometry specimens. Higher rank means a larger 4–6 eV to 6–8 eV spectral-area ratio. The order is not constrained by the inverse-mean-energy rule of the reference model, and no condition-specific calibration factor is introduced to reconcile the two.

S12. Window-integral SR workflow and reference-model comparison

The operational workflow comprises recovery of an energy-referenced RFEA profile, integration over the fixed 4–6 eV and 6–8 eV intervals, calculation of their ratio, and descending-score comparison. Main-text Eq. (1) defines the complementary mean-energy descriptor, and Eq. (2) defines the reported window SR. The original mean-energy reference formulas are preserved in main-text Eqs. (3)–(4) and Eqs. (S7)–(S8) below. Figure 1 illustrates the diagnostic concept and that reference branch. Film measurements provide the complementary process criteria described in Sections S7–S9.

The operational window index, mean-energy response approximation and positive-width reference branch have different inputs. Section S12.3 gives the window calculation used for the seven recorded scores. Section S12.2 retains the mean-energy approximation and its assumptions. Equation (S1) reproduces the analytical curves in Section S5. Equations (S2)–(S5) provide the broader profile-integral and exposure-weighting description. These formulations are distinguished by their definitions, and are not required to produce identical rankings.

S12.1. Acquisition records and descriptor extraction

Each input must link one process condition to its collector-current/retarding-potential record and accepted energy conversion. Retain the run or scan identifier, source configuration, pressure and exposure, source-file checksum, voltage reference, background treatment and analyser-response convention. Ordered source powers identify the condition; they are not converted into mean ion energy. The current transients in Section S3 cannot replace a retarding-potential sweep.

For reconstruction from collector-current sweeps, Eq. (S6) specifies a general profile-recovery relation using the documented energy coordinate E, current I, background current Ibg, sign convention s and positive transmission function T. The relation states the response treatment needed to interpret g_i as a flux-weighted energy profile. It is retained as the acquisition-to-profile model, while the operational SR is calculated from the recovered profile rather than directly from a current-transient mean.

$$g_i(E) = -\frac{s_i}{T_i(E)} \frac{\mathrm{d}\left[I_i(E) - I_{\mathrm{bg},i}(E)\right]}{\mathrm{d}E} \quad \text{(S6)}$$

The energy axis is supplied in eV using the documented potential reference and voltage-to-energy conversion. Derivatives retain the supplied increasing energy grid. Negative recovered-profile values are flagged for review without automatic clipping. Any smoothing, background or transmission treatment must be documented with the accepted input profile.

For an accepted nonnegative profile, energy integrals retain the recorded energy spacing. Mean energy follows main-text Eq. (1), and profile SD is the square root of the second central moment over the same range Ω. A reproducible descriptor implementation can use trapezoidal integration and linear interpolation at the half-height crossings of a single connected peak. FWHM is unresolved if a crossing is outside the recorded range or the half-height regions are disconnected. These descriptor calculations are distinct from the two-window integration specified in Section S12.3.

$$\sigma_i = \sqrt{\frac{\int_{\Omega} (E - \mu_i)^2 g_i(E) dE}{\int_{\Omega} g_i(E) dE}}$$

Mean energy, profile SD and FWHM have energy units. The profile-normalisation area must be positive. Width describes the energy spread within a profile and is not the standard error of its mean. Mean and width are complementary descriptors; neither replaces the two interval areas used in the operational SR.

S12.2. Mean-energy reference model and normalisation

The central relation assumes an effective response factor k_j for each channel j, with j = alkyl or backbone. A_j is a positive response prefactor, and $E_{a,j}$ is the assigned activation-energy parameter. The two factors use the same response convention and are evaluated at the positive mean energy μ_i. The assumed form is given below.

$$k_j(\mu_i) = A_j exp(\frac{-E_{a,j}}{\mu_i})$$

Taking the alkyl factor divided by the backbone factor yields Eq. (S7), with C equal to the alkyl-to-backbone prefactor ratio. Mean ion energy serves as an effective response scale in this phenomenological approximation, rather than a measured material temperature. Across compared conditions, the central model assumes a fixed effective ion population, comparable surface response, a common positive C and a common activation-energy difference. These assumptions define the separate reference-model comparison; nominal source power alone does not establish them.

$$\mathrm{SR}_{\mathrm{model},i} = C\exp\left(\frac{\Delta E_{\mathrm{a}}}{\mu_i}\right) \qquad \text{(S7)}$$

ΔEa is the difference between the assigned backbone and alkyl activation energies. Ref. [16] uses 6.4 and 2.8 eV, giving 3.6 eV for that literature scenario. These activation energies are parameters of the reference approximation. They are not the boundaries of the operational integration windows, and they are not fitted to the seven SR scores. The separate reference-model template requires its own parameter record and does not supply a default value for C.

The activation-energy difference and mean energy use the same units, so the exponent is dimensionless. Choosing C = 1 defines a score normalisation for an analytical scenario. It does not determine a material-specific prefactor ratio. Likewise, the literature value of 3.6 eV is a declared scenario parameter and is not fitted from the present XPS, AFM, optical or profilometry measurements.

$$R_i = \frac{\mathrm{SR}_{\mathrm{model},i}}{\mathrm{SR}_{\mathrm{model},r}} = \exp\left[\Delta E_{\mathrm{a}}\left(\frac{1}{\mu_i} - \frac{1}{\mu_r}\right)\right] \qquad \text{(S8)}$$

Equation (S8) cancels C when both C and the activation-energy difference are shared across compared conditions. Ri denotes the reference-normalised mean-energy model score. The reference-model template uses S50 as a placeholder and requires a corresponding accepted profile before evaluation. This normalisation is retained for the theoretical comparison and was not used to rescale the seven window scores. If a window score is divided by a common positive reference window score, its numerical order is likewise unchanged, but that quotient is not Eq. (S8).

For positive ΔE_{a} and common C, Eq. (S7) is monotonic in mean energy. Its ranking is conditional on that single descriptor and the reference-model assumptions, and is not the ranking rule for the reported window scores. Profile SD and FWHM remain separate descriptors. The Gaussian conversion FWHM/2.355 is not applied automatically to an arbitrary measured shape, and a distribution width is not a standard error of mean energy. The source positive-width branch in Section S5 is a different declared scenario, not an uncertainty distribution for Eq. (S7).

S12.3. Fixed-window integration and seven-condition ranking

The SR column was calculated using the ratio of integrated IEDF intensity in 4–6 eV to that in 6–8 eV. Writing $SR_{win,i}$ to distinguish it from the mean-energy reference score gives the operational definition below, identical to main-text Eq. (2). The same limits apply to every condition.

$$SR_{win,i} = \frac{\int_4^6 g_i(E)\,dE}{\int_6^8 g_i(E)\,dE} \qquad \text{(S9)}$$

With the unit interval weights defined in Section S10.1, Eq. (S9) is the fixed-window special case of Eqs. (S3)–(S4). The profile may be used in its original intensity scale or normalised over Ω, because the common factor cancels. The two integrals must use the same energy reference and response treatment. Neither a higher source power nor a smaller whole-profile mean alone fixes their ratio.

For comparison only, the original pairwise identity below follows from the exponential reference model in Eq. (S7) when its prefactor and activation-energy difference are shared. It is retained to show the mathematical distinction from the window calculation.

$$ln(\frac{SR_{model,i}}{SR_{model,j}}) = \Delta E_a(\frac{1}{\mu_i} - \frac{1}{\mu_j})$$

In that reference model, a shared positive activation-energy difference gives a larger score at a smaller mean energy. Equal means are tied. A common positive prefactor or reference score does not change that order. A zero activation-energy difference gives ties, and a negative difference reverses the direction. These properties apply to Eq. (S7), not to Eq. (S9): two profiles with equal means can have different window ratios.

For $SR_{win,i}$, sort the finite, resolved scores in descending order. A higher score means greater lower-window area relative to upper-window area under the fixed weighting assumption. Equal scores are tied. Both areas should be retained when recomputing the index so that weak denominator signals can be assessed. The ratio does not report total ion dose, total removal, or the intensity above 8 eV. These quantities therefore remain outside the ranking criterion.

The seven recorded values and their original filename labels are listed in Table S2 and ordered in Section S11. Plotting reads the existing SR field without a second SR calculation. The separate reference implementation uses identifiers S50, S100, S150, D50_50, D100_50, D150_50 and D50_100 for ordered source settings. Those identifiers must be linked to the corresponding acquisition records before a new calculation; they do not automatically map the historical filename labels to the profilometry specimens.

For independent numerical reimplementation, use an energy-referenced nonnegative profile covering both complete windows. Piecewise-linear interpolation can insert the 4, 6 and 8 eV boundaries, followed by trapezoidal integration with the actual grid spacing within each interval. The common 6 eV boundary has zero measure in the continuous integral and must not be counted as a duplicated finite-width bin. An incomplete window or an unresolved denominator yields no numerical score. This is an explicit reimplementation specification, not a claim that the original processing code has been recovered or that the seven archived scores have been independently recomputed.

The earlier implementation record reports ten Python tests for descriptor extraction, analytical width, truncated and multimodal profiles, current differentiation, reference normalisation, prefactors, scenario-dependent rank reversal and batch input checks. Those tests use artificial fixtures and concern the separate descriptor-model implementation. They do not verify the historical two-window calculation, and execution of the corresponding MATLAB relative-SR function was not included in that record.

The fixed-window definition supplies the ranking rule. Stability to energy-reference shifts, alternative window boundaries or measurement variation is a separate question. Any such analysis must apply the same perturbation convention to every condition and retain the two component areas. A deterministic sensitivity range has no statistical

confidence level. The width-sensitivity scenarios for the exponential reference model do not estimate uncertainty in the window ratio.

S12.4. Interpretation with the measured film responses

Window-integral SR remains the screening index in the research workflow. Sections S7–S9 supply the accompanying material-response criteria through profilometry, temporal optical evolution, XPS composition and AFM topography. At 100 Pa for 100 s, dual-beam treatment combined greater mean O/C with near-baseline mean roughness, whereas single-beam treatment produced the larger and more variable roughness response. The separate 50 Pa, 180 s series showed no uniform increase in apparent step height under superposition. These measurements are interpreted within their protocols and are not used to fit the window boundaries or to force the numerical SR order. They support preliminary process-window evaluation; direct molecular-selectivity validation would require independently resolved alkyl and backbone responses under matched conditions.

References

References cited in this Supplementary Information follow the numbering of the main manuscript.

[16] Wang, W.; et al. Machine learning-driven soft plasma etching for precision structuring of biofunctional organic semiconductor films. *J. Mater. Chem. C* **2025**, *13*, 22591–22605. DOI: 10.1039/d5tc02791h.

[55] van de Ven, T.H.M.; de Meijere, C.A.; van der Horst, R.M.; van Kampen, M.; Banine, V.Y.; Beckers, J. Analysis of retarding field energy analyzer transmission by simulation of ion trajectories. *Rev. Sci. Instrum.* **2018**, *89*, 043501. DOI: 10.1063/1.5018269.
